# Epigenetic Modulations in Activated Cells Early after HIV-1 Infection and Their Possible Functional Consequences

**DOI:** 10.1371/journal.pone.0119234

**Published:** 2015-04-13

**Authors:** Juliana T. Maricato, Maria N. Furtado, Maisa C. Takenaka, Edsel R. M. Nunes, Patricia Fincatti, Fabiana M. Meliso, Ismael D. C. G. da Silva, Miriam G. Jasiulionis, Maria Cecília de Araripe Sucupira, Ricardo Sobhie Diaz, Luiz M. R. Janini

**Affiliations:** 1 Department of Microbiology, Immunology and Parasitology, Paulista Medical School, Federal University of São Paulo, São Paulo, Brazil; 2 Department of Pharmacology, Federal University of São Paulo, São Paulo, Brazil; 3 Department of Medicine, Federal University of São Paulo, São Paulo, Brazil; International Centre for Genetic Engineering and Biotechnology, ITALY

## Abstract

Epigenetic modifications refer to a number of biological processes which alter the structure of chromatin and its transcriptional activity such as DNA methylation and histone post-translational processing. Studies have tried to elucidate how the viral genome and its products are affected by epigenetic modifications imposed by cell machinery and how it affects the ability of the virus to either, replicate and produce a viable progeny or be driven to latency. The purpose of this study was to evaluate epigenetic modifications in PBMCs and CD4^+^ cells after HIV-1 infection analyzing three approaches: (i) global DNA- methylation; (ii) qPCR array and (iii) western blot. HIV-1 infection led to methylation increases in the cellular DNA regardless the activation status of PBMCs. The analysis of H3K9me3 and H3K27me3 suggested a trend towards transcriptional repression in activated cells after HIV-1 infection. Using a qPCR array, we detected genes related to epigenetic processes highly modulated in activated HIV-1 infected cells. SETDB2 and RSK2 transcripts showed highest up-regulation levels. SETDB2 signaling is related to transcriptional silencing while RSK2 is related to either silencing or activation of gene expression depending on the signaling pathway triggered down-stream. In addition, activated cells infected by HIV-1 showed lower CD69 expression and a decrease of IL-2, IFN-γ and metabolism-related factors transcripts indicating a possible functional consequence towards global transcriptional repression found in HIV-1 infected cells. Conversely, based on epigenetic markers studied here, non-stimulated cells infected by HIV-1, showed signs of global transcriptional activation. Our results suggest that HIV-1 infection exerts epigenetic modulations in activated cells that may lead these cells to transcriptional repression with important functional consequences. Moreover, non-stimulated cells seem to increase gene transcription after HIV-1 infection. Based on these observations, it is possible to speculate that the outcome of viral infections may be influenced by the cellular activation status at the moment of infection.

## Introduction

The term “epigenetic modifications” refers to a number of molecular changes such as DNA-methylation and histones post-translational modifications that, together with chromatin remodeling complexes, nuclear architecture and non-coding RNAs define the structure of chromatin and its transcriptional activity [1,2]. These modifications, although not involving changes in the DNA sequence, can alter gene expression. Epigenetic modulations occur in response to several environmental stimuli, such as behavioral, physiological, and pathological, and are inherited and reversible [3–7].

Epigenetic modifications can occur at three levels: (i) directly over the DNA, such as methylation of CpG islands; (ii) at the transcriptional/translational level by modulating the expression of proteins, which are responsible for performing epigenetic changes; and (iii) at the post-translational level, such as modification of histones and other DNA-associated proteins exemplified by acetylation, deacetylation, and methylation. While these are the main epigenetic modifications, many of them are still poorly understood, probably because the cellular epigenetics study is relatively recent [3,4].

One of the focuses on epigenetics research related to Human Immunodeficiency Virus type 1 (HIV-1) infections is to elucidate how the viral genome and their protein products may be affected by differential methylation or histone modification, and how this affects the ability of the virus to infect and produce viable progenies. In addition, it has been observed that epigenetic modulations may induce the virus to remain latent and integrated in host cell genome, helping the establishment of a viral reservoir difficult to be accessed with regular antiretroviral drugs.

Recent study showed that viral proteins undergo methylation, which can affect the viral progeny production. Treatment of transfected or infected cells with methylation inhibitors decreased viral production showing that methylation helped to promote the fusion between the viral envelope and the cell membrane, suggesting a relationship between methylation and infectivity [8].

Other studies have demonstrated that the HIV-1 genome, once integrated into the host DNA, undergoes transcriptional repression due to epigenetic modifications [9–11]. These modifications may result in viral latency in CD4^+^ T cells and the consequent maintenance of a latent reservoir even under highly active antiretroviral therapy [9].Non-selective inhibitors of histone deacetylases have been used for therapeutic purposes in HIV-1 positive patients [10]. These inhibitors are capable to induce proviral expression, disrupting latency in order to expose the virus to antiretroviral therapy [10,11].

Virus entry can elicit several changes in the infected cells, which could be either a cellular response to the virus or the activation of still unknown viral functions over the host cell [12–15]. Recent studies addressing virus and epigenetics indicate that viruses can modulate the host cell epigenetic machinery to control its replication and to repress viral restriction. Furthermore, it has been demonstrated that several types of viral infections are able to induce epigenetics changes in host cells, controlling viral activation and latency [16–19].

In this context, one study addressed the effect of HIV-1 on epigenetic marks in CD8^+^ T cells and showed that, during HIV-1 infection, these cells lost their antiviral functions due to the expression and signaling through of the inhibitory programmed death-1 receptor (PD-1). They observed that the PD-1 promoter remained unmethylated in HIV-specific CD8^+^ T cells from treated subjects with suppressed viral loads or from elite controllers [13].

The HIV-1-induced epigenetic modulations were also demonstrated in the CD4^+^ FoxP3^+^ regulatory T cells (Treg) [14]. Recent study showed that Treg cells suffered demethylation in the FOXP3 promoter after HIV-1 infection. Furthermore, an elevated frequency of Treg cells was found in the gut mucosa of HIV-1 infected patients due to possible aberrant methylation processes [14].

Moreover, it has been shown that during infections in central nervous system (CNS), HIV-1 may interfere with BCL11B and other chromatin modifiers related to gene silencing. Alteration of epigenetic factors might result in abnormal transcriptomes, leading to inflammation, neurodegeneration, neurocognitive impairment and deregulation of proinflammatory cytokines [15].

On the other hand, it is also possible to hypothesize that, under HIV-1 infection, the host cell changes its epigenetic pattern by “recognizing” an incoming viral particle in order to protect itself from a potentially lethal infection.

The present study aimed to investigate whether the early stages of HIV-1 infections could lead to changes in cellular epigenetic patterns including increased methylation and post-translational modifications of histones. Also, we verified, how early these modifications can occur and how they might affect functional features of cells.

The evaluation of epigenetic modifications was done through the analysis of the global DNA methylation, and of classical epigenetic markers such as H3K9, H3K27 and H3K4 trimethylation. In addition, we carried on a quantitative array RNA analyzing the expression levels of 84 genes related to epigenetic modifications.

Based on our findings, we were able to observe: (i) significant epigenetic modulations occurring in primary PBMCs and CD4^+^ T cells during the early stages of HIV-1 infection (in the first 36 hours during cell cultures) despite the cellular activation status at the moment of viral infection; (ii) opposite trends towards the modulation of epigenetic mechanisms between activated and non-activated cells at 24 hours after HIV-1 infection and (iii) a pattern indicative of transcriptional repression in activated host-cells early after HIV-1 infection including the down regulation of activation markers, Interleukin-2 (IL-2), Interferon- γ (INF-γ) and metabolism-related factors.

## Materials and Methods

### Ethics Statement

We would like to express our sincere gratitude to Dr. Flávia Latini and COLSAN—Associação Beneficente de Coleta de Sangue who contributed to this study providing health donor’s blood samples.

### Cell Culture, stimulus and flow cytometry analysis

Peripheral blood mononuclear cells (PBMCs) were obtained from blood of healthy, hepatitis/HIV-1-seronegative donors by Ficoll-hypaque (GE Healthcare) density gradient according manufacturer’s instructions. In some experiments CD4+ T cells were purified from PBMCs using EasySep Human CD4 Positive Selection Kit (Stem Cell). PBMCs or purified CD4^+^ T cells were cultured in RPMI medium containing 10% FBS, 2mM de L-glutamine (Invitrogen), 100 U/mL penicillin and 100 mg/mL streptomycin (Invitrogen).

Cells were stimulated with 10 μg/mL of Phytohemagglutinin (PHA) (Invitrogen) and 20 U/mL of IL-2 (Invitrogen) for 48h followed by 24h incubation with only IL-2. After this period, cells were stained with anti-CD3, anti-CD4, anti-CD25, anti-CD69 and anti-HLA-DR antibodies and the cellular activation was analyzed by flow cytometry [20]. PBMCs without stimulus (non-activated cells) were used as control.

The cell activation was analyzed in the gate of CD3^+^CD4^+^ T cells. Data were analyzed by using the FlowJo Program (Tree Star Inc.). Fifty thousand events were acquired on a FACSCalibur flow cytometer (BD Biosciences).

### *In vitro* HIV-1 infection

Virus stocks used were produced from the pNL4-3 molecular infectious clone (cat. num. 114, AIDS Reagent Program, NIH, United States). Activated or non-activated cells were infected with HIV-1 at a multiplicity of infection (MOI) of 0.05 for 2 h. Cells were washed three times with warmed RPMI to remove residual free viral particles and maintained in culture 6, 12, 24 or 36 hours (h). After these periods, supernatants were removed and cells were harvested for RNA, DNA and protein extraction, which were used in further experiments. Non-activated cells were analyzed only at 24h after HIV-1 infection.

### Global 5-methylcytosine levels in the cellular genome

Total DNA from each experimental condition was extracted from 2x10^6^ infected or non-infected PBMCs using the Trizol (Invitrogen) method according manufacturer’s instructions. Samples from three different infections obtained from cells from three different donors were pooled to obtain 6 μg of genomic DNA to perform a single experiment with a single technical replicate. In other words, cells from three different healthy donors were collected, PBMCs from each donor were separately infected with HIV-1 for 6h, 12h, 24h, and 36h and genomic DNA were recovered after the indicated time-points. At each time-point, genomic DNA samples from the different donors were pooled constituting one biological sample (we obtained an amount of two biological samples in this study). Each biological sample had its DNA digested with the restriction enzymes mentioned below.

To analyze the global 5’-methylcytosine, 1 μg of genomic DNA from pooled samples was digested using the restriction enzymes HpaII (which digests only targets with non-methylated cytosines—at the motif—CCGG) and MspI (which digests targets with both non-methylated and methylated cytosines at the motif—CC(CH3)pGG) [21,22]. After 16–18h of digestion at 37°C, the samples were submitted to electrophoresis in 0.8% agarose gels stained with SybrSafe dye (Invitrogen) at the concentration of 1:10.000. Images were acquired using an UV transilluminator with DigiDoc-It Systems (UVP) and analyzed with the software ImageJ v. 1.45s (Public domain, NIH, USA). To calculate the global 5’-methylcytosine levels, band intensities were normalized by subtracting the background intensity from each acquired image. The percentage of methylated genomic DNA was obtained using the following formula: % of DNA methylation = the HpaII digested band intensity—the MspI digested band intensity X100/ the non-digested genomic DNA band intensity [21–23].

At least three measurements of the band intensity were made for each experimental condition to generate the plotted values. All the experiments were performed in biological duplicates.

### Total RNA extraction

RNA samples from 2x10^7^ PBMCs or purified CD4^+^ T cells from each experimental condition were obtained using the RNAeasy Micro Kit (Qiagen) following the manufacturer’s protocol. The RNA integrity was verified by electrophoresis in 1% agarose gel stained with SybrSafe dye (Invitrogen) at a concentration of 1:10.000.

### Quantitative real-time PCR (qPCR) array of genes involved in epigenetic modifications

To perform one single experiment samples from three different infections with cells from three different healthy donors were pooled as described above to constitute which we called one biological sample.

280 ng of total RNA from each biological sample were used as input in a reaction to synthesize cDNAs using the RT2 First Strand kit (SA Biosciences) according to the *RT² Profiler PCR Array* (SA Biosciences) protocol [24].

Gene expression analysis of chromatin modification enzymes was performed using the *RT² Profiler PCR Array Human Epigenetic Chromatin Modification Enzymes kit* (PAHS-085A, SA Biosciences). This array allowed the expression analysis of 84 genes that encode for enzymes related to chromatin remodeling, histone post-translational modification, and DNA methylation. It also analyzed the expression of other co-relate enzymes, which participate in chromatin assessment and, therefore, in the cellular gene expression. The complete table containing the description of all genes evaluated in this array can be found at Table A in S1 Dataset.

Quantitative real-time PCR assays were performed according SA Biosciences’ guidelines [24]. Reactions were carried out in the *ABI Prism 7500 Sequence Detector System* (Applied Biosystems, Life Technologies.).

Calculations of gene expression and statistics were made following the manufacturer’s tutorial available at http://sabiosciences.com/ pcrarraydataanalysis.php. Relative quantification of gene expressions was established by comparing the Relative Fluorescent Units and Cycle threshold values (Cts) for each studied gene with the ones obtained from control genes (housekeeping genes) using the ΔΔCt method described previously [25,26].

Gene expression modulations were considered of statistical significance when fold change values were equal or greater than threefold. Thus, positive regulation (up-regulation) was considered significant when fold change values were ≥ 3 and negative regulation (down-regulation) was significant when fold change values were ≤ -3.

### Quantitative real-time PCR (qPCR) of individual genes

280 ng of RNA from CD4^+^ T cells from each experimental condition derived from healthy donors were submitted to complementary DNA (cDNA) synthesis with SuperScript II reverse transcriptase kit (Life Technologies) according to the manufacturer’s protocol.

Gene expression was evaluated by qPCR using the SYBR Green-based system of detection (Applied Biosystems, Life Technologies). Briefly, each reaction was composed of 2 μM of forward and reverse oligonucleotides for each target gene or GAPDH as endogenous control, 10 μl of the Power SYBR Green PCR master mix (Applied Biosystems) and 3 μl of cDNA diluted 2.5×. Cycling reactions were carried out in the Applied Biosystems 7500 System (Applied Biosystems) starting with one cycle of 50°C (2 min) and 95°C (1min), followed by 45 cycles at 95°C (15 s) and 60°C (1 min). Melting curves were determined with an additional cycle of 95°C (15 s), 60°C (20 s) and 95°C (15 s).

To quantify the gene expression, the ΔΔCt method of relative quantification was used to compare the expression levels of the same transcript in different samples. As recommended, standardization reactions were carried out to evaluate the efficiency of amplification for each gene tested. Efficiencies of amplification were calculated using the equation E = (10 − 1/slope)× 100 and only reactions that had efficiency from 90% to 100% were accepted [25,26]. Reactions were performed in triplicates. For each time point the non-infected group was used as reference. Oligonucleotides sequences used were: GAPDH-F (5’ GTCTCCTCTGACTTCAACAGCG 3’, GAPDH-R (5' ACCACCCTGTTGCTGTAGCCAA 3’), IL-2-F (5' AGAACTCAAACCTCTGGAGGAAG 3'), IL-2 (5' GCTGTCTCATCAGCATATTCACAC 3'), IFN-γ-F (5’ TTTCATGCCTGGTGCTTCCA 3’), IFN-γ-R (5’ GCTAAGAAGACTCCCCTCCCT 3’), Gys-1-F (5' CCGCTATGAGTTCTCCAACAAGG 3'), Gys-1-R (5' AGAAGGCAACCACTGTCTGCTC 3'), Glut-1-F (5' TTGCAGGCTTCTCCAACTGGAC 3'), Glut-1-R (5' CAGAACCAGGAGCACAGTGAAG 3'), Eno-1-F (5' TTGCAGGCTTCTCCAACTGGAC 3'), Eno-1-R (5' CAGAACCAGGAGCACAGTGAAG 3'), SETDB2-F (5’ GTTTCCTCGGAGTCTGTCACTC 3’), SETDB2-R (5’ CAACATGGTTTGAACTTGAGTCCT 3’), RSK2-F (5’ CGAGGTCATACTCAGAGTGCTG 3’), RSK2-R (5’ ACTGTGGCATTCCAAGTTTGGCT 3’).

### Western blot analysis

Protein extractions from each experimental condition were obtained from 2x10^7^ PBMCs or purified CD4^+^ T cells by adding 200 μL of RIPA Buffer and 2 μL of 100x Halt Phosphatase and Protease Inhibitor Cocktails (Thermo Scientific). The resulting mixtures were sonicated for 3 cycles of 60 sec at 30% of amplitude and 20 sec of pause in the sonicator LB-130FSJ (Labometric). Total protein extracts were quantified using the BCA method with the BCA Assay kit (Pierce).

To perform each single Western blot, protein extracts experiment samples consisting of three different infections with cells from three different donors were pooled as described above.

The levels of trimethylation of histone H3 at lysine 9 (H3K9me3), trimethylation of histone H3 at lysine 27 (H3K27me3); trimethylation of histone H3 at lysine 4 (H3K4me3) were detected in pooled samples by using specific antibodies (Millipore). The antibody anti-H3 was used as control (Millipore). Reactions were developed with the enhanced Super Signal Chemiluminescence kit (Pierce) at the photo-illuminator Alliance 4.7 (UVITEC) and the images were analyzed using the UVIBand software (UVITEC).

To measure post-translational modification levels on global H3, band intensities were normalized by subtracting the background intensity for each acquired image. The percentage of methylation on lysines 9, 27 and 4 on H3 was obtained using the following formula: % of H3 methylation = the H3K9 or H3K4 or H3K27 band intensity x100/ the H3 band intensity (normalizer) [22]. Statistical analyzes were performed comparing control samples (non-infected cells) with tested samples (infected cells) for each time-point (paired analysis).

SETDB2 and RSK2 protein levels were detected in pooled samples by using specific antibodies (Millipore). The antibody anti-GAPDH was used as a control (Thermo Fischer). Reactions were developed with the enhanced Super Signal Chemiluminescence kit (Pierce) at the photo-illuminator Alliance 4.7 (UVITEC) and the images were analyzed using the UVI Band software (UVITEC). The ratios between specific protein bands and GAPDH were calculated and statistical analyzes were performed comparing control samples (non-infected cells) with tested samples (infected cells) for each time-point (paired analysis). At least three measurements of the band intensity were made for each experimental condition to generate the plotted values. All the experiments were performed in biological duplicates.

### Statistical analysis

Data are presented as means ± standard deviations (SD). Data were analyzed by unpaired Student’s t test with Welch’s correction (two-tailed), which is used for comparison of two groups when the data meet the assumptions of the t tests. Analysis of variance (ANOVA) followed by the Bonferroni test was applied.

## Results

### PBMC activation status prior to HIV-1 infection

**Fig 1:**
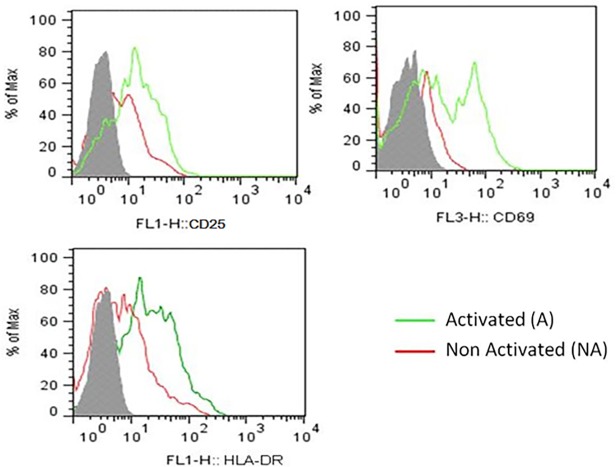
Activation status of the PBMCs prior to HIV-1 infection. PBMCs were gated on CD3^+^CD4^+^ and stained for the activation markers CD25 **(upper panel—right)**, CD69 **(upper panel—left)** and HLA-DR **(lower panel)**. Activated cells (green lines), non-activated cells (red lines) and unstained control (gray peaks). This histogram is representative of six independent experiments.

To analyze the impact of the HIV-1 on the epigenetics of activated PBMCs, we firstly activated these cells and the cellular activation status before viral infection was verified by flow cytometry. As expected, the activation markers CD25, CD69, and HLA-DR were up-regulated on CD3^+^CD4^+^ T cells upon stimulation (Fig. 1). Next, we evaluated epigenetic modifications in activated PBMCs-HIV-1 infected at 6, 12, 24 and 36h after virus infection. We also analyzed the epigenetic modifications in non-activated PBMCs infected by HIV-1 at 24h post-infection. The HIV-1 infection efficiency in activated- and non-activated-PBMCs was evaluated through the presence of viral DNA by qPCR. We found that activated- PBMCs contained higher levels of proviral DNA than non-activated-PBMCs. The number of viral DNA was 12.3 and 28.8 copies per 10^6^ cells at 24 and 36h post-infection in activated-PBMCs, respectively (S1 Fig.—dark bars). In contrast, in non-activated-PBMCs the proviral load was 3.9 copies at 24h and 13.1 copies at 36h after HIV-1 infection (S1 Fig.—white bars). These results are consistent with data described the in literature showing that unstimulated cells are less permissive to HIV-1 infection and that the viral cycle in these cells is delayed in relation to stimulated cells [20,27–29].

### Analysis of epigenetic modifications

#### Analysis of global 5’-methylcytosine in the cellular genome of HIV-1 infected PBMCs

The first epigenetic modification evaluated was the global content of 5’-methylcytosines in the cellular genomic DNA from each experimental group using the methodology of genomic DNA digestion with Msp I and Hpa II restriction enzymes.

**Fig 2:**
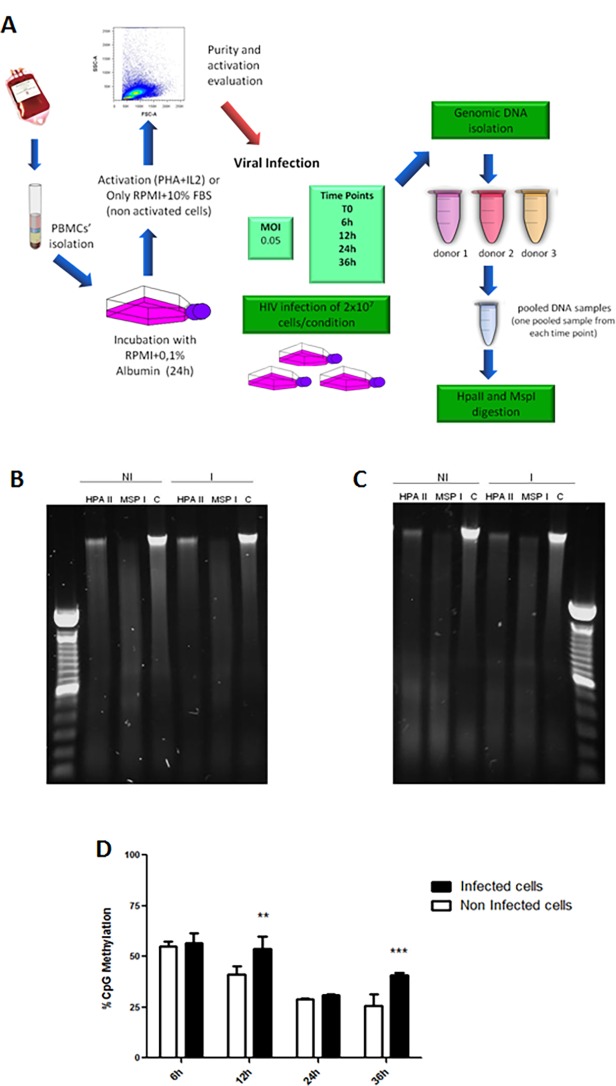
Experimental design and global DNA Methylation levels in HIV-1 infected activated PBMCs. **(A)** Schematic representation of the experimental design and conditions: PBMC from healthy donors were separated by Ficoll gradient (in some experiments CD4^+^ cells were purified by positive selection). Cell viability was accessed by Trypan Blue exclusion. Cells were cultivated with a poor medium (RPMI+0.1% Bovine Albumin) for 24h in order to minimize any undesired previous activation. Next, cells were washed and resuspended in RPMI (supplemented)+10%FBS and activation stimulus (PHA+IL2) was added (activated group) or left without activation stimulus (non-activated group). After 36 hours of stimulation, the purity and activation status of cells were checked by flow cytometry using common human CD4^+^ T cell activation markers (CD25, HLA-DR and CD69). Next, 2x10^7^ cells were infected with HIV at a MOI (multiplicity of infection) of 0.05. The infections were carried out during the indicated time intervals (6h, 12h, 24h or 36h). After infection periods, cells were harvested, washed with PBS and cellular pellets were submitted to genomic DNA and RNA and protein extraction. **(B) and (C)** Experimental duplicates of 36h time-point agarose gel electrophoresis of genomic DNA from PBMCs after digestion with restriction enzymes HpaII and MspI. Gels were stained with SybrSafe dye (1:10.000—Invitrogen). HPA II—genomic DNA digested with Hpa II; MSP I genomic DNA digested with Msp I; C- Non-digested genomic DNA (Control). (**NI**) DNA from non-infected cells, (**I**) DNA from HIV-1 infected cells. The images are a representative of an experimental duplicate in which the cells were collected at 36h after HIV-1 infection. The experiments were performed in biological duplicates. **(D)** Percentage of 5’-methylcytosine content in genomic DNA at different time points. Dark bars—HIV-1 infected cells; White bars—non-infected cells. The data represent the mean of three different measurements and the error bars indicate the differences between three independent experiments. 2way ANOVA: *** p< 0.001, ** p < 0.01 and *, p < 0.05.

Our goal was to describe an epigenetic pattern in response to early viral infection rather than looking at the variability that exists in the methylation of cells derived from individuals. To address this question, we opted to work with pooled infected cells instead of individual specific samples. The assessment of global 5’-methylcytosine levels was performed here according to the schematic representation shown in Fig. 2A.

Fig. 2B and 2C show two representative images of agarose gels containing the whole genomic DNA digestion with the enzymes MspI and HpaII. These images were acquired for band density analysis and are representative of two biological replicates of extracted DNA 36h after viral infection.

The analysis of global 5’-methylcytosine in the genomic DNA from each experimental group showed a higher percentage of methylation in the HIV-1 infected cells when compared with non-infected cells at 12 and 36h after infection (Fig. 2D). Interestingly, the period of about 36h hours after infection, roughly represents the time necessary for the completion of one viral cycle. However, at 6h after viral infection the percentage of methylation are not significantly different between infected and uninfected cells, possibly reflecting there is period required for the mobilization of cellular functions promoting epigenetic changes after viral infection. Taken together, these results indicate that HIV-1 mediated a transcriptional repression in infected cells.

#### Expression levels of genes involved in epigenetic modifications after HIV-1 infection

The next step was to determine changes in the expression of genes involved with the modification of chromatin and other epigenetic mechanisms in the HIV-1 infected cells and non-infected cells using an array of 84 genes encoding enzymes and proteins that participate in cellular epigenetic modifications, including methyltransferases, histone acetylases and deacetylases.

The first set of data was generated from 12 gene-expression arrays. Results obtained from all infected cells were compared to results from all non-infected cells. This analysis allowed us to determinate which genes were modulated after HIV-1 infection regardless the time point. By doing this, it was possible to restrict the number of modulated genes to those that were mostly modulated during HIV-1 infection (Table 1).

**Table 1:** Gene expression modulations after HIV-1 infection—modulated genes comparing infected cells versus non-infected cells (control group).

RefSeq	Gene	Description	2^-ΔC_t_		T-TEST	Fold Up- or Down-Regulation
			Infected	Non-infected	p value	Infected /Non-infected
NM_019854	PRMT8	Protein arginine methyltransferase 8	6,9E-05	2,4E-04	0,0903415	-**3,54**
NM_004586	RPS6KA3	Ribosomal protein S6 kinase, 90kDa, polypeptide 3	2,2E+00	5,9E-02	0,141151	**+36,84**
NM_024860	SETD6	SET domain containing 6	7,3E-03	2,5E-02	0,112047	-**3,39**
NM_031915	SETDB2	SET domain, bifurcated 2	6,8E+00	5,6E-02	0,141115	**+121,30**

After the first analysis, we performed a paired analysis in which we compared each infected sample to its respective non-infected control. With this approach, it was possible to identify which genes were modulated at each time point after HIV-1 infection (Tables A to F in S1 Dataset).

**Fig 3:**
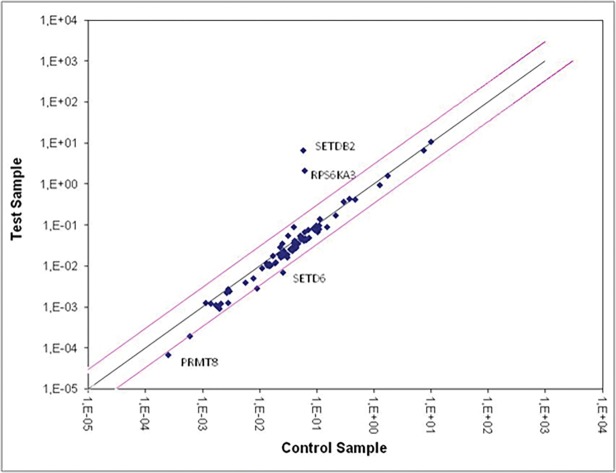
Scatter plot showing expression levels of the 84 genes encoding for enzymes related to epigenetic modifications. The diagram represents the relative expression levels calculated in log (2^-ΔΔCt^) for each one of the 84 genes (blue dots). The data was generated using the RT² Profiler PCR Array Human Epigenetic Chromatin Modification Enzymes (PAHS-085A, SA Biosciences). The analysis was performed comparing the data from uninfected cells at 6, 12, 24 and 36h (x-axis) versus infected cells (y-axis) at 6, 12, 24 and 36h after infection. Pink lines delimit the zone of change in gene expression between −3 and +3 fold. The central axis (dark blue line) represents the mean normalized endogenous control expression. Dots above the pink lines correspond to up-regulated genes and bellow the line to down-regulated genes. Genes showing up-regulation ≥ +3 and down-regulation ≤ −3 are indicated in the plot.

Table 1 and Fig. 3 show the most relevant results from the analysis of infected cells versus non-infected cells regardless the time point. We found that the genes PRMT8, RPS6KA3, STED6 and SETDB2 were differentially expressed in HIV-1 infected cells when compared with their uninfected controls. Moreover, we observed that the fold-changes for up-regulated genes were higher than the fold-changes for down-regulated genes (Table 1).

When we analyzed the changes in gene expression along time of infection, we observed that HIV infection induced an intense gene expression modulation in all time points, mainly within 24 hours post infection (Table 2, S2 Fig.). Cells showing a higher variation in gene expression were the non-activated infected cells at 24 hours post-infection, indicating that the cellular activation status at the moment of viral infection interferes with the expression of some. For a better understanding of these results, we listed the most modulated genes (up and down-regulated) by experimental condition. We listed only genes that underwent modulation in, at least, in two conditions. Using this criterion it was possible to establish a set of 11 genes, here considered the most important in terms of epigenetic regulation in the HIV-1 infected cells when compared with its respective controls (absence of infection). We found that genes under the highest modulation, that is, detected in more than one condition, was the gene coding for the ribosomal protein S6 kinase polypeptide 3 (RPS6KA3, also known as RSK2), an important kinase that is activated by the MAPK pathway and the gene coding for the SET domain bifurcated 2 (SETDB2) involved in several epigenetic modifications as discussed below. These results led us to choose RSK2 and SETDB2 for further study.

**Table 2:** The most modulated genes in HIV-1 infected cells from 6h to 36h hours after infection.

RefSeq	Gene	Description	6h	12h	24h	24h NA	36h
NM_004586	RPS6KA3	Ribosomal protein S6 kinase, 90kDa, polypeptide 3	UM	UM	up	up	UM
NM_031915	SETDB2	SET domain, bifurcated 2	UM	UM	up	up	UM
NM_019854	PRMT8	Protein arginine methyltransferase 8	down	UM	UM	down	UM
NM_024860	SETD6	SET domain containing 6	UM	UM	UM	down	down
NM_001379	DNMT1	DNA (cytosine-5-)-methyltransferase 1	UM	up	up	down	UM
NM_005933	MLL	Myeloid/lymphoid or mixed-lineage leukemia (trithorax homolog, Drosophila)	UM	up	UM	down	up
NM_003160	AURKC	Aurora kinase C	UM	up	UM	down	UM
NM_004824	CDYL	Chromodomain protein, Y-like	UM	up	UM	down	UM
NM_014648	DZIP3	DAZ interacting protein 3, zinc finger	down	UM	UM	down	UM
NM_024827	HDAC11	Histone deacetylase 11	UM	UM	Up	down	UM
NM_012330	KAT6B	K(lysine) acetyltransferase 6B	UM	UM	down	down	UM

Gene expression fold changes comparing infected versus non-infected cells. Up: up-regulated genes; down—down-regulated genes; UM—unmodulated genes.

We also found that histone deacetylases and methyltransferases genes were up-regulated in activated PBMCs post-HIV-1 infection (Tables A to D in S1 Dataset). This is consistent with the increase of CpGs methylation detected in activated PBMCs infected with HIV-1 (Fig. 2—B, C and D). Overall, these results taken together support the hypothesis that activated PBMCs undergo transcriptional repression, after HIV-1infection.

To confirm the positive modulation of RSK2 and SETDB2 in HIV-1 infected cells detected by qPCR array, we analyzed the RSK2 and SETDB2 mRNA expression using conventional qPCR. We also investigated the protein levels of RSK2 and SETDB2 by Western Blot using pools of infected- or non-infected-CD4^+^ T cells obtained from PBMCs of healthy donors. Pools were prepared as indicated in Fig. 2A. We found an up-regulation of RSK2 gene expression in the CD4^+^ T cells at the earliest time-points post-HIV-1 infection, being more evident at 24 hours post infection (S2 Fig.—A). On the other hand, protein levels of RSK2 increased at 6h and 12h after HIV-1 infection, and decreased at 24h with a different kinetic when compared to the transcriptional kinetic (S2 Fig.—A and C). For the SETDB2 mRNA, we observed a significant increase after HIV-1 infection at 6h, 24h and 36h time-points (S2 Fig.—B) and the protein production increased over the time in infected cells with statistical significance at the 24h time-point (S2 Fig.-D). Altogether, these data show increased expression of RSK2 and SETDB2 at transcriptional and translational levels in host cells after HIV-1 infection.

#### Western blot analysis of post-translational histone modifications in activated cells after HIV-1 infection

Our previous results indicated that activated infected cells underwent modulation of genes coding for proteins and enzymes involved in epigenetic mechanisms. In order to verify these alterations we checked the status of some epigenetic markers by Western blot. We evaluated in infected activated PBMCs the percentage of trimethylation occurring on lysines K27 and K9 of the histone H3 (H3K27me3 and H3K9me3 respectively), which are classic markers of epigenetic transcriptional silencing in eukaryotes. The percentage of trimethylation of the lysine 4 in the histone H3 (H3K4me3), a classic marker for epigenetic transcriptional activation, was also analyzed [4].

**Fig 4:**
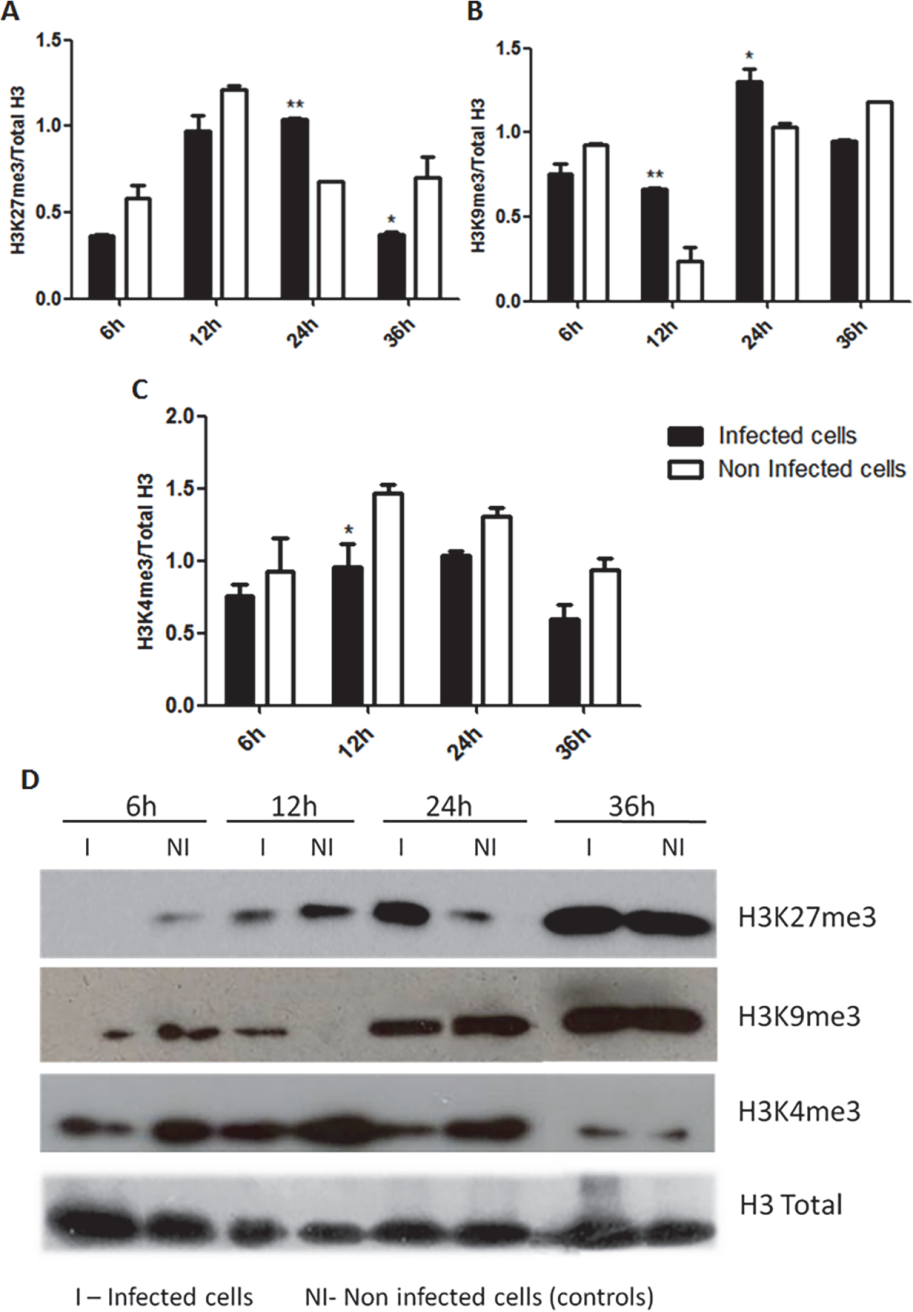
Classical markers of epigenetic transcriptional silencing and activation in activated PBMCs after HIV-1 infection. **(A)** Graphical representation of protein ratios of epigenetic transcriptional silencing marker H3K27me3 over the total H3. **(B)** Graphical representation of protein ratios of epigenetic transcriptional silencing marker and H3K9m3 over the total H3. **(C)** Graphical representation of protein ratios of epigenetic transcriptional activation marker H3K4m3 over the total H3. Protein levels of each marker were calculated by the ratio of band intensities between specific markers (H3K27me3, H3K9me3 or H3K4me3) over the total H3 (normalizer) using the software ImageJ v. 1.45s (Public domain, NIH, USA). Dark bars—HIV-1 infected cells; White bars—non-infected cells (control group). **(D)** Representative Western blot image for each epigenetic marker (H3K27me3—upper panel, H3K9me3—middle panel, H3K4me3—lower panel and the total H3 as normalizer. The data represent the mean of three different measurements of the same experiment and the error bars indicate the differences between two independent experiments. 2way ANOVA: *** p< 0.001, ** p < 0.01 and *, p < 0.05. (**NI**) non-infected cells, (**I**) HIV-1 infected cells.

Fig. 4A and 4B show the ratio in band intensities of H3K27me3 and H3K9me3, respectively, over the total H3 intensity. Fig. 4C depicts the ratio in band intensities of H3K4me3 over the total H3 intensity. In Fig. 4B, we observed an increase in the ratios of silencing marker H3K9me3 over the total H3 when compared to non-infected cells in all time-points. In addition, H3K27m3 levels are up-regulated at 12h and 24h in infected-cells (Fig. 4A). These results are suggestive of a global decrease in the cellular transcriptional activity after HIV-1 infection, especially at 12 and 24h time-points. Moreover, it was possible to note lower ratios of H3K4me3/H3 total in activated infected PBMCs (Fig. 4C and 4D). Together, these results support our hypothesis that, from the global perspective, a reduction in the transcriptional activity might be occurring in these cells.

### Possible functional consequences of epigenetic modulations in activated cells after HIV-1 infection

**Fig 5:**
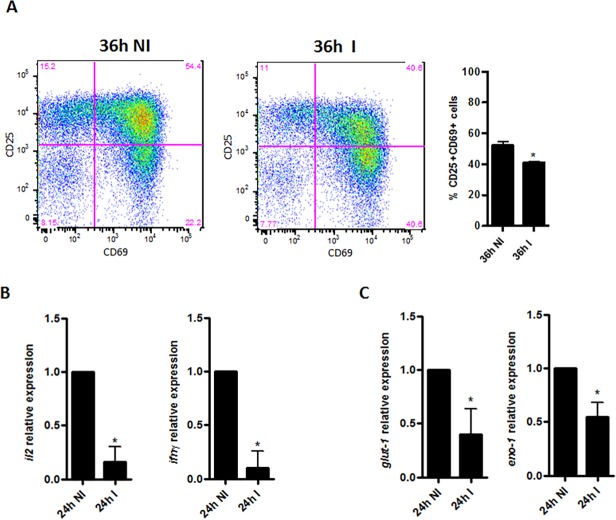
Modulation in the functional markers of activated CD4+ T cells after HIV-1 infection. **(A)** Flow cytometry of purified CD4^+^ T cells at 36h post HIV-1 infection—dot plots of cell populations (gated on CD4^+^CD3^+^ cells) analyzed for the T cell early activation markers CD25, CD69 (percentages are shown in each quadrant) and graphical representation of the percentages of CD25^+^CD69^+^ cells (gated on CD4^+^CD3^+^ cells). **(B)** IL2 and IFN- γ mRNA relative expression in CD4+ T cells at 24h after HIV-1 infection. **(C)** GLUT-1 and ENO-1 mRNA relative expression in CD4+ T cells at 24h after HIV-1 infection. GAPDH was used as a normalizer of all reactions to calculate the relative expression by 2^-ΔΔCt^ method. Data are shown as mean ± SD of triplicates and are representative of three independent experiments using cells of three different healthy donors. Two-tailed Student’s t-test: *, p < 0.05. (**NI**) non-infected cells, (**I**) HIV-1 infected cells.

To evaluate if the global repression of transcriptional activity mediated by HIV-1 infection in activated cells may impact on the cell function, we purified CD4^+^ T cells from PBMCs of healthy donors and, after *in vitro* HIV-1 infection, we evaluated three important parameters for CD4^+^ T cells functional activity (Fig. 5). Thus, the T cell activation was analyzed through the surface expression of the main important early activation markers CD25 and CD69, which are involved with cell proliferation and signaling [20,30].

We observed a decrease in the frequency of CD25^+^CD69^+^CD4^+^ T cells after HIV-1 infection when compared with non-infected cells (Fig. 5A). Accordingly, we also observed a reduced levels of IL-2 and INF-γ transcripts in infected cells (Fig. 5B), which are two important factors for cell survival and maintenance of immune response, respectively [31].

Recently, some studies have been reported the association between metabolism-related genes and functional features of immune cells, including the CD4^+^ T cells [32,33]. Loisel-Meyer and colleagues have demonstrated that Glu-1 (Glucose transporter 1) an enhancer glycolysis factor, which is a downstream substrate of AKT/PI3K pathway, can regulate HIV-1 infection in CD4^+^ T cells [33]. We evaluated the gene expression of Glut-1 and Eno-1 (alpha-enolase-1) another important factor for the glycolysis pathway. Surprisingly, we found a reduction of about 50% in the expression of transcripts for Glu-1 and Eno-1 in infected TCD4^+^ cells (Fig. 5C).

Thus, in activated cells at early moments post HIV-1 infection, we found a predominance of global epigenetic modulations related to transcriptional repression and, as a possible consequence, we detected the down-regulation in the expression of genes of extreme importance for the functional activity of CD4^+^ T cells.

### Cellular activation status prior to HIV-1 infection might interfere with epigenetic modulations

**Fig 6:**
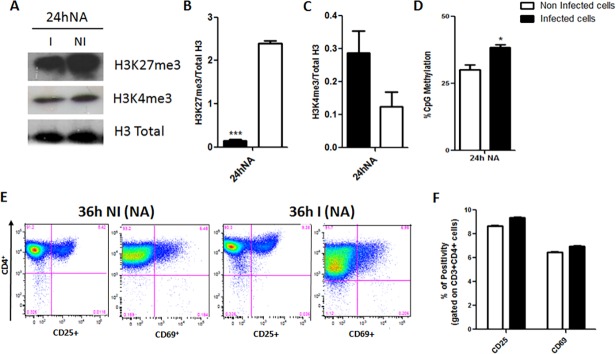
Classical markers of epigenetic transcriptional silencing and activation of non-activated PBMCs at 24h after HIV-1 infection. **(A)** Representative Western blot image for each epigenetic marker (H3K27me3—upper panel and H3K4me3—lower panel) and the total H3 as normalizer. **(B)** Graphical representation of protein ratios of epigenetic transcriptional silencing marker and H3K27m3 over the total H3. **(C)** Graphical representation of protein ratios of epigenetic transcriptional activation marker H3K4m3 over the total H3. Protein levels of each marker were calculated by the ratio of band intensities between specific markers (H3K27me3, H3K9me3 or H3K4me3) over the total H3 (normalizer) using the software ImageJ v. 1.45s (Public domain, NIH, USA). Dark bars—HIV-1 infected cells. **(D)** Percentage of 5’-methylcytosine content in genomic DNA. Data represent the mean of three different measurements of the same experiment and the error bars indicate the differences between two independent experiments. 2way ANOVA: *** p< 0.001, ** p < 0.01 and *, p < 0.05. **(E)** Flow cytometry of non-activated purified CD4^+^ T cells 36h post HIV-1 infection—dot plots of cell populations (gated on CD4^+^CD3^+^ cells) analyzed for the T cell early activation markers CD25, CD69 (percentages are shown in each quadrant) and graphical representation of the percentages of CD25^+^CD69^+^ cells (gated on CD4^+^CD3^+^ cells). Data are shown as mean ± SD of triplicates and are representative of three independent experiments using cells of three different healthy donors. Two-tailed Student’s t-test: *, p < 0.05. Dark bars—HIV-1 infected cells, White bars—non-infected cells, NA—non-activated cells. (**NI**) non-infected cells, (**I**) HIV-1 infected cells.

In order to verify whether the cellular activation status may interfere with the epigenetic modulations observed in this study, we investigated the levels of the epigenetic markers H3K27me3, H3K4me3 and the global levels of genomic CpG-methylated in non-activated cells that are cells that did not receive activation stimulus prior to HIV-1 infection (Fig. 6).

Since most of the significant changes in activated cells infected by HIV-1 occurred at the 24h post-infection, a time point where the virus should have already completed the reverse transcription step and integrated the viral DNA in the cellular genome [20,28,34,35], we decided to analyze the same parameters in infected, but non-activated, PBMCs populations at the 24h after infection (Fig. 6 Tables 1, 2 and Table E in S1 Dataset). Comparing the most modulated genes between activated and non-activated cells at 24h post-infection (Tables D and E in S1 Dataset), we observed that SETDB2 and RPS6KA3 were up-regulated in both conditions, however in activated cells the modulation occurred to a greater extent. In addition, in non-activated cells, factors related to transcriptional repression, such as histone deacetylases and methyltransferases genes, were down-regulated. The same was not observed in activated cells 24h post-infection (Tables B, C, D and F in S1 Dataset).

In non-activated cells, at 24h post HIV-1 infection, we observed a significant decrease of the H3K27me3/Total H3 ratio of infected cells comparing to the uninfected control (Fig. 6 - A and B). Surprisingly, when we analyzed the transcriptional activation marker H3K4m3 over the total H3 we found increased levels in HIV-1 infected group of non-activated cells (Fig. 6 - A C), revealing opposite profiles between activated and non-activated cells at 24h after HIV-1 infection. These results may indicate that an increase in transcriptional activity is taking place in non-activated cells. However, the percentage of global methylation of genomic DNA is still significantly higher in these cells elapsed at 24h after viral infection than in uninfected controls (Fig. 6D) following a similar pattern to what we found for the activated cells (Fig. 2D).

We also evaluate the percentages of the early activation markers CD25 and CD69 in these cells at 36h post HIV-1 infection. Fig. 6E and 6F show that at this time point there is no significant alterations in the frequency of CD25^+^CD69^+^CD4^+^ population between infected and non-infected groups, demonstrating that HIV-1 infection appears to not influence these surface markers expression after 36h post-infection, the later time-point addressed in the present study.

In summary, we conclude that the activation status of the cell before HIV-1 infection might interfere qualitatively in the epigenetic modulations that occur in response to the viral infection.

## Discussion

Recent studies on HIV-1 and epigenetics have tried to elucidate how the viral genome and its products may be affected and cellular epigenetic mechanisms and how this could interfere with the processes of infection, viral replication and production of viral progeny. Other publications have addressed how the viral products and cellular proteins may contribute to virus integration and its latency in order to establish a reservoir that may be activated later during the infection [16–19].

Here, we investigated the influence of HIV-1 infections in the host’s epigenetic mechanisms during the first 36 hours of infection that is considered the average time of one single cycle of viral replication [20,27,35].

We evaluated the modulation of epigenetic mechanisms in HIV-1 infected cells at three different levels: (i) directly on the DNA, by measuring the 5’-methylcytosine content; (ii) at the transcriptional/translational level by modulating the expression of genes that encode proteins responsible for epigenetic changes that occur directly to the DNA or other proteins, and (iii) at the post-translational level, by measuring the methylation of classical epigenetic markers for transcriptional silencing and activation.

We analyzed the impact of HIV-1 infection on the increased global DNA methylation of cells that could indicate a tendency for transcriptional repression in activated cells early after HIV-1 infection. Previous studies reported that methylation of CpG islands associated with the integrated viral genome could also represent also a strategy used by the HIV-1 to maintain viral latency [13]. Furthermore, it was experimentally demonstrated that methylation of two CpG islands flanking the transcription initiation site of HIV-1 promoters are critical for viral latency in infected cells [29]. Moreover, some findings in the literature showed that enzymes such as MBD2 and histone deacetylase 2 (HDAC2) can bind to these islands during latent viral infection and that, the treatment of the latent infected-cells with 5-aza 2'deoxycytidine-(aza-CdR), a demethylating agent, inhibited the recruitment of MBD2 and HDAC2 to the viral transcription initiation site causing the reactivation of latent viruses [36].

Another study has shown that, depending on the level of methylation on the HIV-1 promoter, the latency may be easily reversed. In patients with undetectable plasma viremia promoters and enhancers of HIV-1 were hypermethylated and resistant to reactivation, while in viremic patients, the same region of the HIV-1 genome was hypomethylated, establishing a direct correlation between percentage of methylation and latency in host cells [19]. However, a recent study from Siliciano’s group, demonstrated that antiretroviral therapy fails to eradicate HIV-1 infection because latent proviruses persist in resting CD4+ T cells despite the low levels of CpG methylation in LTRs [37]. Moreover, the same research group found that current latency-reversing agents (LRAs), including demethylation agents, were not fully able to reactivate HIV-1 latency *in vivo* and are unlikely to eliminate the *in vivo* latent reservoir [34].

Thus, the results presented here allowed us to speculate two hypotheses: (i) After infection, HIV-1 might interfere with the cellular environment in order to become latent and evade the host’s immune response; (ii) the global transcriptional reduction observed could be a cellular response to minimize the viral success during its integration into the host genome. Some findings in the literature support ours second hypotheses: Wang et al. have been demonstrated the “preference” for viral integration in non-methylated chromatin in which there is a relevant transcriptional activity [11].

Next, we investigated, in infected cells, the expression of several genes encoding proteins and enzymes that participate in epigenetic mechanisms. This approach allowed us to better understand the effect of HIV-1 infection over the modulation of genes related to epigenetic control driving host cells to either silencing or activation of transcription. Among the up-regulated genes detected after HIV-1 infection, the gene encoding for SETDB2 protein had the most up-regulated expression. The SETDB2 protein is capable of modulating gene expression by interfering with epigenetic mechanisms such as histone H3 methylation. SETDB2 is a histone H3 methyltransferase and contains both the active site and the flanking cysteine residues required for the catalytic activity of this class of enzymes [38–40]. Histone H3-methylations are classic epigenetic markers, which can increase gene transcription when lysines K4, K36 or K79 are trimethylated. Moreover, they can also induce gene silencing when the trimethylation occurs at lysines K9 and K27 [4,41]. Our results indicated that in infected cells, mainly at 24 hours after HIV-1 infection, the expression of SETDB2 is up-regulated in either activated or non-activated cells, indicating that HIV-1 infection could be correlated with the expression of this gene. Thus, SETDB2 up-regulation could be considered an earlier marker of the HIV-1 infection in host cells. SETDB2 can influence either the transcription or silencing of other genes depending on the lysine of H3 that is trimethylated [38]. This issue is being addressed in more detail by other investigators in our research group.

RPS6KA3, also known as RSK2, is another important gene that we found up-regulated in HIV-1-infected cells. We identified an important up-regulation of RSK2 at 24h after HIV-1 infection in both activated and non-activated cells. However, RSK2 up-regulation occurred in a less extent in non-activated cells. To our knowledge, there is yet no report correlating RSK2 expression and HIV-1 infection. RSK2 gene encodes for a protein of the same name, which is a member of the subfamily of ribosomal S6 kinases, belonging to the family of serine/threonine protein kinases that contains two catalytic domains and are kinases that phosphorylate several substrates in mammals including the via of mitogen-activated kinases (MAPK) [39]. The main activity of these proteins is related to cell growth and differentiation [40–42]. Interesting, in both activated and non-activated infected cells, RSK2 expression was up-regulated. On the other hand, we found that important genes for transcriptional silencing as methyltransferases and histone deacetylases (i.e. HDAC2) were down-regulated in non-activated cells but, up-regulated in activated cells after HIV-1 infection. Furthermore, our results showed that, elapsed 24h after HIV-1 infection, activated and non-activated cells infected displayed antagonistic modulation for the epigenetic transcriptional markers H3K27me3 and H3K4me3 associated with transcriptional repression and transcriptional activation, respectively.

Since, even along the immune responses, the lymphocytic cell population comprises both activated and non-activated cells, taken together, these observations might indicate a bimodal viral strategy in its interaction with host cells trying to induce viral production for the establishment of a persistent infection in non-activated cells and, on the other way, trying to preserve the viral genetic material towards to induction of transcriptional repression which might lead activated cells to the latency establishment in order create HIV-1 reservoirs.

Regarding the post-translational alterations of the classical epigenetic transcriptional markers evaluated in the present study, we observed a strong tendency for transcriptional repression in activated cells between 12h and 24h after HIV-1 infection. These data are consistent with the global CpG methylation profile found for these cells. Nevertheless, the inversion in the ratios of H3K27me3 and H3K9me3 over the total H3 observed in activated infected cells at 6h and 36h after infection can be explained by the fact that at 6h after viral infection was probably too soon for epigenetic modulations at the protein level were probably too early, although, alterations in cellular gene expression were observed as soon as 6h after HIV-1 infection (Table B in S1 Dataset). According to the HIV-1 replication cycle in activated cells, 36h is the time needed for the virus to complete its cycle. It is already known that HIV-1 takes 1.47 days since its entry into the cell until production of the viral progeny [20,27,35]. We speculate that the alteration in epigenetic profiles seen at 36h after infection may represent a viral strategy to increase transcription in order to optimize the completion of the virus cycle and viral egress. In summary, our global epigenetic analysis on activated cells at early moments after HIV-1 infection revealed a tendency for transcriptional repression.

Thus, our next step was to investigate whether this tendency for transcriptional repression would be related to some functional consequence for these cells. First, we analyzed the expression of the early activation surface markers CD25 and CD69 in the activated cells at 36h after HIV-1 infection. We found a significant decrease in the frequency of CD25^+^CD69^+^CD4^+^ cell population. Since, these important early activation markers are involved in cell proliferation and signaling and it is known that the viral protein Nef interacts with CD3 signaling in Jurkat cells to down-regulate CD69 expression [20,30], we could speculated that this interaction with activated cells may occur through the modulation of epigenetic mechanisms. The fact that non-activated cells display a different epigenetic profile from that found for activated cells at 24h post HIV-1 infection and that the frequency of CD25^+^CD69^+^ CD4^+^ T cell population is altered by the presence of HIV-1 at the same time point studied for activated cells (36h) reinforces our hypothesis.

We also verified the levels of IL-2 and INF-γ, two important cytokines for cell survival and maintenance of the immune response, respectively, [31], in activated cells and found them down-regulated mainly at 24h post-infection. These two cytokines are of extreme importance for T helper cell orchestration of the adaptive phase of the immune responses against HIV-1 and other pathogens and are constantly evaluated in clinical trials when the response to potential HIV-1 vaccines is addressed. [43,44].

To address whether the possible functional impairment in the HIV-1 infected activated cells could be related to some metabolic feature, we investigated the expression of two glycolysis pathway genes: Glu-1 and Eno-1, supported by the recent findings that glycolysis metabolism genes, such as Glut-1, are essential for CD4^+^ T cell activation and effector functions [32]. Moreover, by increasing Glut-1 expression, quiescent CD4^+^ T cells become more susceptible to HIV-1 infection and down-regulation of Glut-1 abrogates the infection. [33]. Our results show decreased levels of Glut-1 and Eno-1 mRNAs in activated cells, principally at 24h post HIV-1 infection suggesting that HIV-1 infection may negatively affects important metabolic processes in the host cells.

Thus, in activated cells at early moments post HIV-1 infection, we found a predominance of global epigenetic modulations related to transcriptional repression and, as a possible functional consequence, we detected down-regulation in the main surface activation markers as well as in expression of genes of extreme importance for the functional activity of CD4+ T cells.

In the present work we showed that cells infected by HIV-1 exhibit modulations at the genomic, transcriptional and protein level. Thus, HIV-1 appears to interfere with the cellular epigenetic regulation leading to distinct profiles of histone modifications and/or inducing the expression of enzymes, such as DNA methyltransferases, HATs, HDACs and others. By interfering with epigenetic mechanisms that regulate gene expression and protein production, HIV-1 might act to modulate host cells in different manners depending on the intracellular environment. Consistent with this, preliminary results showed that the cellular activation state may influence the epigenetic modulation that is triggered after HIV-1 infection, which underscores the plasticity of this virus trying to adapt to different cellular environments. Nonetheless, additional experiments are needed to clarify and possibly confirm these hypotheses.

Moreover, because of the intriguing results found, new studies are already ongoing in our research group to expand this investigation addressing these and other epigenetic modifications in non-activated cells at several time-points after viral infection.

In summary, the viral interference may disturb host cell environment resulting in functional consequences, such as modulation of epigenetic machinery, effector cytokines, metabolic enzymes, signal transducing pathways and other key factors that maintain the essential balance for optimal survival of the cell.

## Supporting Information

S1 Fig Absolute quantitation of Proviral Load in PBMCs along 36h of HIV-1 infection. Quantitative assay was performed using a TaqMan (Life Sciences) test for the HIV-1 integrase gene as target and the human CCR5 gene as normalizer. The presence of viral DNA was expressed as absolute copy numbers/10^6^ cells. Time points are indicated in the x-axis. C+—Activated PMBCs at 72h after HIV-1 infection, C- Non-infected activated PBMCs cultured for 72h. White bars—non-activated PBMCs; Dark bars—Activated PBMCs. Data are shown as mean ± SD of triplicates and are representative of three independent experiments using cells of three different pooled samples. Two-tailed Student’s t-test: *, p < 0.05.
(TIF)

S2 Fig Evaluation of transcriptional and proteic modulation of RSK2 and SETDB2 in activated CD4+ T cells along 36h hours of HIV-1 infection. **(A)** RSK2 mRNA relative expression in activated CD4+ T cells after HIV-1 infection. **(B)** SETDB2 mRNA relative expression in activated CD4^+^ T cells after HIV-1 infection. Gapdh was used as normalize of all reactions to calculate relative expression by 2^-ΔΔCt^ method. Data are shown as mean ± SD of triplicates and are representative of three independent experiments using cells of three different healthy donors. Two-tailed Student’s t-test: *, p < 0.05. **(C)** Representative Western blot image for RSK2 and GAPDH as normalize (upper panel) and graphical representation of protein ratios of RSK2 over GAPDH (lower panel). **(D)** Representative Western blot image for SETDB2 and GAPDH as normalize (upper panel) and graphical representation of protein ratios of SETDB2 over GAPDH (lower panel). Protein levels were calculated by the ratio of band intensities between specific protein over GAPDH (normalizer) using the software ImageJ v. 1.45s (Public domain, NIH, USA). The data represent the mean of three different measurements of the same experiment and the error bars indicate the differences between two independent experiments. 2way ANOVA: *** p< 0.001, ** p < 0.01 and *, p < 0.05. (**NI**) non-infected cells, (**I**) HIV-1 infected cells.
(TIF)

S1 Dataset Supplemental Tables from A to F. **Table A**, List of all genes studied in RT² Profiler PCR Array Human Epigenetic Chromatin Modification Enzymes. **Table B,** List of modulated genes comparing infected cells versus non-infected cells (control group) at 6h time-point. **Table C,** List of modulated genes comparing infected cells versus non-infected cells (control group) at 12h time-point. **Table D,** List of modulated genes comparing infected cells versus non-infected cells (control group) at 24h Activated time-point. **Table E,** List of modulated genes comparing infected cells versus non-infected cells (control group) at 24h Non Activated time-point. **Table F,** List of modulated genes comparing infected cells versus non-infected cells (control group) at 36h time-point.
(DOC)

S1 Methods Methos and References for quantification of HIV-1 proviral loads. (DOC)
