# Supplementary figures and images for: Epigenetic Modulations in Activated Cells Early after HIV-1 Infection and Their Possible Functional Consequences
Source: PLoS One. 2015 Apr 13;10(4):e0119234. doi: 10.1371/journal.pone.0119234 (PMC4395311; doi:10.1371/journal.pone.0119234)

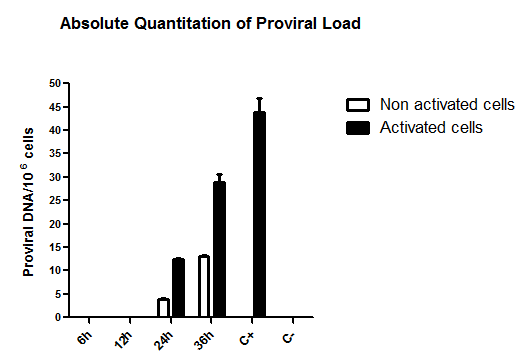

Supplement: S1 Fig — Quantitative assay was performed using a TaqMan (Life Sciences) test for the HIV-1 integrase gene as target and the human CCR5 gene as normalizer. The presence of viral DNA was expressed as absolute copy numbers/106 cells. Time points are indicated in the x-axis. C+—Activated PMBCs at 72h after HIV-1 infection, C- Non-infected activated PBMCs cultured for 72h. White bars—non-activated PBMCs; Dark bars—Activated PBMCs. Data are shown as mean ± SD of triplicates and are representative of three independent experiments using cells of three different pooled samples. Two-tailed Student’s t-test: *, p < 0.05. (TIF) [file pone.0119234.s001.tif]

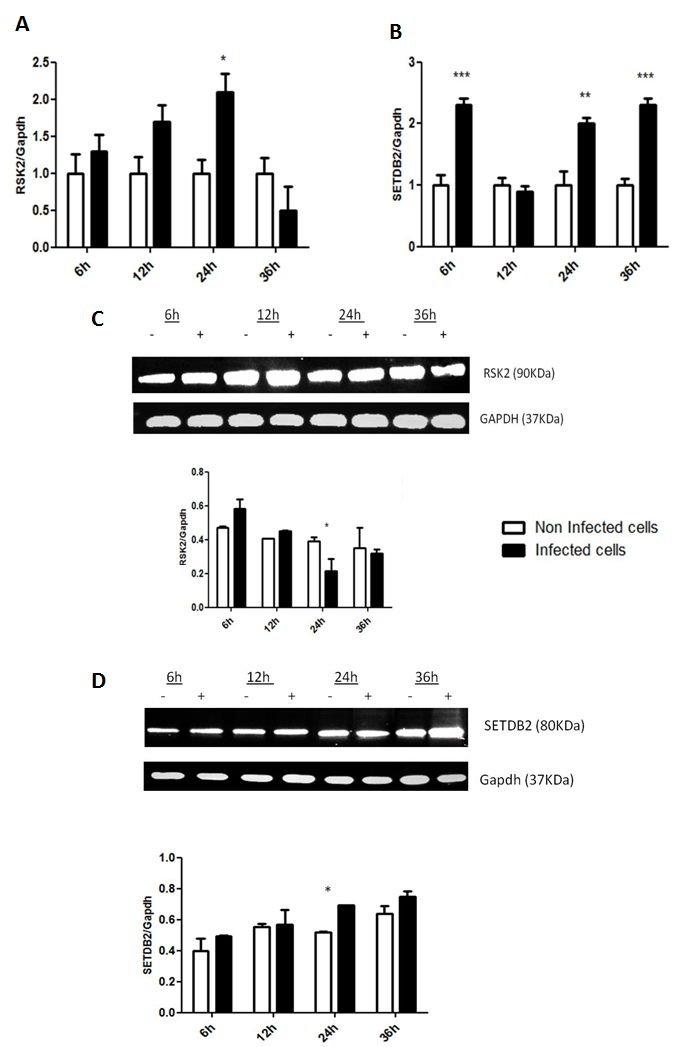

Supplement: S2 Fig — (A) RSK2 mRNA relative expression in activated CD4+ T cells after HIV-1 infection. (B) SETDB2 mRNA relative expression in activated CD4+ T cells after HIV-1 infection. Gapdh was used as normalize of all reactions to calculate relative expression by 2-ΔΔCt method. Data are shown as mean ± SD of triplicates and are representative of three independent experiments using cells of three different healthy donors. Two-tailed Student’s t-test: *, p < 0.05. (C) Representative Western blot image for RSK2 and GAPDH as normalize (upper panel) and graphical representation of protein ratios of RSK2 over GAPDH (lower panel). (D) Representative Western blot image for SETDB2 and GAPDH as normalize (upper panel) and graphical representation of protein ratios of SETDB2 over GAPDH (lower panel). Protein levels were calculated by the ratio of band intensities between specific protein over GAPDH (normalizer) using the software ImageJ v. 1.45s (Public domain, NIH, USA). The data represent the mean of three different measurements of the same experiment and the error bars indicate the differences between two independent experiments. 2way ANOVA: *** p< 0.001, ** p < 0.01 and *, p < 0.05. (NI) non-infected cells, (I) HIV-1 infected cells. (TIF) [file pone.0119234.s002.tif]
