# Supplementary material for: Epigenetic Modulations in Activated Cells Early after HIV-1 Infection and Their Possible Functional Consequences
Source: PLoS One. 2015 Apr 13;10(4):e0119234. doi: 10.1371/journal.pone.0119234 (PMC4395311; doi:10.1371/journal.pone.0119234)
Supplement: S1 Dataset — Table A, List of all genes studied in RT² Profiler PCR Array Human Epigenetic Chromatin Modification Enzymes. Table B, List of modulated genes comparing infected cells versus non-infected cells (control group) at 6h time-point. Table C, List of modulated genes comparing infected cells versus non-infected cells (control group) at 12h time-point. Table D, List of modulated genes comparing infected cells versus non-infected cells (control group) at 24h Activated time-point. Table E, List of modulated genes comparing infected cells versus non-infected cells (control group) at 24h Non Activated time-point. Table F, List of modulated genes comparing infected cells versus non-infected cells (control group) at 36h time-point. (DOC) [file pone.0119234.s003.doc]

**Supplemental Tables**

**Table A –** List of all genes studied in RT² Profiler PCR Array Human Epigenetic Chromatin Modification Enzymes.

| **Position** | **UniGene** | **RefSeq** | **Symbol** | **Description** | **Gene Name** |
| --- | --- | --- | --- | --- | --- |
| A01 | Hs.591518 | NM_015013 | KDM1A | Lysine (K)-specific demethylase 1A | AOF2, BHC110, KDM1, KIAA0601, LSD1 |
| A02 | Hs.491060 | NM_018489 | ASH1L | Ash1 (absent, small, or homeotic)-like (Drosophila) | ASH1, ASH1L1, FLJ10504, KIAA1420, KMT2H |
| A03 | Hs.592510 | NM_001880 | ATF2 | Activating transcription factor 2 | CRE-BP1, CREB2, HB16, MGC111558, TREB7 |
| A04 | Hs.250822 | NM_003600 | AURKA | Aurora kinase A | AIK, ARK1, AURA, AURORA2, BTAK, MGC34538, STK15, STK6, STK7 |
| A05 | Hs.442658 | NM_004217 | AURKB | Aurora kinase B | AIK2, AIM-1, AIM1, ARK2, AurB, IPL1, STK12, STK5, aurkb-sv1, aurkb-sv2 |
| A06 | Hs.98338 | NM_003160 | AURKC | Aurora kinase C | AIE2, AIK3, ARK3, AurC, STK13, aurora-C |
| A07 | Hs.371416 | NM_199141 | CARM1 | Coactivator-associated arginine methyltransferase 1 | PRMT4 |
| A08 | Hs.269092 | NM_004824 | CDYL | Chromodomain protein, Y-like | CDYL1, DKFZp586C1622, MGC131936 |
| A09 | Hs.701991 | NM_000246 | CIITA | Class II, major histocompatibility complex, transactivator | C2TA, CIITAIV, MHC2TA, NLRA |
| A10 | Hs.728790 | NM_020536 | CSRP2BP | CSRP2 binding protein | ATAC2, CRP2BP, KAT14, MGC15388, PRO1194, dJ717M23.1 |
| A11 | Hs.202672 | NM_001379 | DNMT1 | DNA (cytosine-5-)-methyltransferase 1 | AIM, CXXC9, DNMT, FLJ16293, MCMT, MGC104992 |
| A12 | Hs.515840 | NM_022552 | DNMT3A | DNA (cytosine-5-)-methyltransferase 3 alpha | DNMT3A2, M.HsaIIIA |
| B01 | Hs.643024 | NM_006892 | DNMT3B | DNA (cytosine-5-)-methyltransferase 3 beta | ICF, M.HsaIIIB |
| B02 | Hs.713641 | NM_032482 | DOT1L | DOT1-like, histone H3 methyltransferase (S. cerevisiae) | DKFZp586P1823, DOT1, KIAA1814, KMT4 |
| B03 | Hs.409210 | NM_014648 | DZIP3 | DAZ interacting protein 3, zinc finger | FLJ13327, FLJ57977, FLJ58022, FLJ58223, KIAA0675, UURF2, hRUL138 |
| B04 | Hs.709218 | NM_006709 | EHMT2 | Euchromatic histone-lysine N-methyltransferase 2 | BAT8, C6orf30, DKFZp686H08213, FLJ35547, G9A, KMT1C, NG36 |
| B05 | Hs.464733 | NM_052911 | ESCO1 | Establishment of cohesion 1 homolog 1 (S. cerevisiae) | A930014I12Rik, CTF, ECO1, EFO1, ESO1, KIAA1911, MGC105022 |
| B06 | Hs.99480 | NM_001017420 | ESCO2 | Establishment of cohesion 1 homolog 2 (S. cerevisiae) | 2410004I17Rik, EFO2, RBS |
| B07 | Hs.632532 | NM_003642 | HAT1 | Histone acetyltransferase 1 | KAT1 |
| B08 | Hs.88556 | NM_004964 | HDAC1 | Histone deacetylase 1 | DKFZp686H12203, GON-10, HD1, RPD3, RPD3L1 |
| B09 | Hs.26593 | NM_032019 | HDAC10 | Histone deacetylase 10 | DKFZp761B039, HD10, MGC149722 |
| B10 | Hs.404802 | NM_024827 | HDAC11 | Histone deacetylase 11 | FLJ22237, HD11 |
| B11 | Hs.3352 | NM_001527 | HDAC2 | Histone deacetylase 2 | HD2, RPD3, YAF1 |
| B12 | Hs.519632 | NM_003883 | HDAC3 | Histone deacetylase 3 | HD3, RPD3, RPD3-2 |
| C01 | Hs.20516 | NM_006037 | HDAC4 | Histone deacetylase 4 | AHO3, BDMR, HA6116, HD4, HDAC-A, HDACA, KIAA0288 |
| C02 | Hs.438782 | NM_005474 | HDAC5 | Histone deacetylase 5 | FLJ90614, HD5, NY-CO-9 |
| C03 | Hs.6764 | NM_006044 | HDAC6 | Histone deacetylase 6 | FLJ16239, HD6 |
| C04 | Hs.200063 | NM_001098416 | HDAC7 | Histone deacetylase 7 | DKFZp586J0917, FLJ99588, HD7A, HDAC7A |
| C05 | Hs.310536 | NM_018486 | HDAC8 | Histone deacetylase 8 | HD8, HDACL1, RPD3 |
| C06 | Hs.196054 | NM_178425 | HDAC9 | Histone deacetylase 9 | DKFZp779K1053, HD7, HD7b, HD9, HDAC, HDAC7, HDAC7B, HDAC9B, HDAC9FL, HDRP, KIAA0744, MITR |
| C07 | Hs.443650 | NM_006618 | KDM5B | Lysine (K)-specific demethylase 5B | CT31, FLJ10538, FLJ12459, FLJ12491, FLJ16281, FLJ23670, JARID1B, PLU-1, PLU1, PUT1, RBBP2H1A |
| C08 | Hs.631768 | NM_004187 | KDM5C | Lysine (K)-specific demethylase 5C | DXS1272E, JARID1C, MRXJ, MRXSJ, SMCX, XE169 |
| C09 | Hs.155983 | NM_014663 | KDM4A | Lysine (K)-specific demethylase 4A | JHDM3A, JMJD2, JMJD2A, KIAA0677 |
| C10 | Hs.709425 | NM_015061 | KDM4C | Lysine (K)-specific demethylase 4C | FLJ25949, GASC1, JHDM3C, JMJD2C, KIAA0780, bA146B14.1 |
| C11 | Hs.223678 | NM_001080424 | KDM6B | Lysine (K)-specific demethylase 6B | JMJD3, KIAA0346 |
| C12 | Hs.463045 | NM_021078 | KAT2A | K(lysine) acetyltransferase 2A | GCN5, GCN5L2, MGC102791, PCAF-b, hGCN5 |
| D01 | Hs.533055 | NM_003884 | KAT2B | K(lysine) acetyltransferase 2B | CAF, P, P, CAF, PCAF |
| D02 | Hs.528299 | NM_006388 | KAT5 | K(lysine) acetyltransferase 5 | ESA1, HTATIP, HTATIP1, PLIP, TIP, TIP60, cPLA2 |
| D03 | Hs.25674 | NM_003927 | MBD2 | Methyl-CpG binding domain protein 2 | DKFZp586O0821, DMTase, NY-CO-41 |
| D04 | Hs.258855 | NM_005933 | MLL | Myeloid/lymphoid or mixed-lineage leukemia (trithorax homolog, Drosophila) | ALL-1, CXXC7, FLJ11783, HRX, HTRX1, KMT2A, MLL, GAS7, MLL1A, TET1-MLL, TRX1 |
| D05 | Hs.647120 | NM_170606 | MLL3 | Myeloid/lymphoid or mixed-lineage leukemia 3 | DKFZp686C08112, FLJ12625, FLJ38309, HALR, KIAA1506, KMT2C, MGC119851, MGC119852, MGC119853 |
| D06 | Hs.592262 | NM_182931 | MLL5 | Myeloid/lymphoid or mixed-lineage leukemia 5 (trithorax homolog, Drosophila) | FLJ10078, FLJ14026, HDCMC04P, KMT2E, MGC70452 |
| D07 | Hs.709264 | NM_001085487 | MYSM1 | Myb-like, SWIRM and MPN domains 1 | 2A-DUB, 2ADUB, DKFZp779J1554, DKFZp779J1721, KIAA1915, RP4-592A1.1 |
| D08 | Hs.533803 | NM_032188 | KAT8 | K(lysine) acetyltransferase 8 | FLJ14040, MOF, MYST1, hMOF |
| D09 | Hs.21907 | NM_007067 | KAT7 | K(lysine) acetyltransferase 7 | HBO1, HBOA, MYST2 |
| D10 | Hs.491577 | NM_006766 | KAT6A | K(lysine) acetyltransferase 6A | MGC167033, MOZ, MYST3, RUNXBP2, ZNF220 |
| D11 | Hs.35758 | NM_012330 | KAT6B | K(lysine) acetyltransferase 6B | DKFZp313G1618, FLJ90335, KIAA0383, MORF, MOZ2, MYST4, qkf, querkopf |
| D12 | Hs.596314 | NM_003743 | NCOA1 | Nuclear receptor coactivator 1 | F-SRC-1, KAT13A, MGC129719, MGC129720, RIP160, SRC1, bHLHe42, bHLHe74 |
| E01 | Hs.592142 | NM_181659 | NCOA3 | Nuclear receptor coactivator 3 | ACTR, AIB-1, AIB1, CAGH16, CTG26, KAT13B, MGC141848, RAC3, SRC-3, SRC3, TNRC14, TNRC16, TRAM-1, bHLHe42, pCIP |
| E02 | Hs.368971 | NM_014071 | NCOA6 | Nuclear receptor coactivator 6 | AIB3, ASC2, KIAA0181, NRC, PRIP, RAP250, TRBP |
| E03 | Hs.197071 | NM_014397 | NEK6 | NIMA (never in mitosis gene a)-related kinase 6 | SID6-1512 |
| E04 | Hs.106861 | NM_022455 | NSD1 | Nuclear receptor binding SET domain protein 1 | ARA267, DKFZp666C163, FLJ10684, FLJ22263, FLJ44628, KMT3B, SOTOS, STO |
| E05 | Hs.435714 | NM_002576 | PAK1 | P21 protein (Cdc42/Rac)-activated kinase 1 | MGC130000, MGC130001, PAKalpha |
| E06 | Hs.20521 | NM_001536 | PRMT1 | Protein arginine methyltransferase 1 | ANM1, HCP1, HRMT1L2, IR1B4 |
| E07 | Hs.154163 | NM_001535 | PRMT2 | Protein arginine methyltransferase 2 | HRMT1L1, MGC111373 |
| E08 | Hs.152337 | NM_005788 | PRMT3 | Protein arginine methyltransferase 3 | HRMT1L3 |
| E09 | Hs.367854 | NM_006109 | PRMT5 | Protein arginine methyltransferase 5 | HRMT1L5, IBP72, JBP1, SKB1, SKB1Hs |
| E10 | Hs.26006 | NM_018137 | PRMT6 | Protein arginine methyltransferase 6 | FLJ10559, FLJ51477, HRMT1L6 |
| E11 | Hs.712584 | NM_019023 | PRMT7 | Protein arginine methyltransferase 7 | FLJ10640, KIAA1933 |
| E12 | Hs.504530 | NM_019854 | PRMT8 | Protein arginine methyltransferase 8 | HRMT1L3, HRMT1L4 |
| F01 | Hs.591490 | NM_007212 | RNF2 | Ring finger protein 2 | BAP-1, BAP1, DING, HIPI3, RING1B, RING2 |
| F02 | Hs.729085 | NM_019592 | RNF20 | Ring finger protein 20 | BRE1, BRE1A, FLJ11189, FLJ20382, KIAA2779, MGC129667, MGC129668, hBRE1 |
| F03 | Hs.445387 | NM_004586 | RPS6KA3 | Ribosomal protein S6 kinase, 90kDa, polypeptide 3 | CLS, HU-3, ISPK-1, MAPKAPK1B, MRX19, RSK, RSK2, S6K-alpha3, p90-RSK2, pp90RSK2 |
| F04 | Hs.510225 | NM_004755 | RPS6KA5 | Ribosomal protein S6 kinase, 90kDa, polypeptide 5 | MGC1911, MSK1, MSPK1, RLPK |
| F05 | Hs.297483 | NM_014712 | SETD1A | SET domain containing 1A | KIAA0339, KMT2F, Set1, Set1A |
| F06 | Hs.507122 | NM_015048 | SETD1B | SET domain containing 1B | FLJ20803, KIAA1076, KMT2G, Set1B |
| F07 | Hs.517941 | NM_014159 | SETD2 | SET domain containing 2 | FLJ16420, FLJ22472, FLJ23184, FLJ45883, FLJ46217, HBP231, HIF-1, HIP-1, HYPB, KIAA1732, KMT3A, SET2, p231HBP |
| F08 | Hs.510407 | NM_199123 | SETD3 | SET domain containing 3 | C14orf154, DKFZp761E1415, FLJ23027, MGC87236 |
| F09 | Hs.606200 | NM_017438 | SETD4 | SET domain containing 4 | C21orf18, C21orf27 |
| F10 | Hs.288164 | NM_001080517 | SETD5 | SET domain containing 5 | DKFZp686J18276, FLJ10707, KIAA1757 |
| F11 | Hs.592060 | NM_024860 | SETD6 | SET domain containing 6 | FLJ21148 |
| F12 | Hs.480792 | NM_030648 | SETD7 | SET domain containing (lysine methyltransferase) 7 | FLJ21193, KIAA1717, KMT7, SET7, SET7, 9, SET9 |
| G01 | Hs.572262 | NM_020382 | SETD8 | SET domain containing (lysine methyltransferase) 8 | KMT5A, PR-Set7, SET07, SET8 |
| G02 | Hs.643565 | NM_012432 | SETDB1 | SET domain, bifurcated 1 | ESET, H3-K9-HMTase4, KG1T, KIAA0067, KMT1E |
| G03 | Hs.631789 | NM_031915 | SETDB2 | SET domain, bifurcated 2 | C13orf4, CLLD8, CLLL8, DKFZp586I0123, DKFZp761J1217, KMT1F |
| G04 | Hs.567571 | NM_022743 | SMYD3 | SET and MYND domain containing 3 | FLJ21080, KMT3E, MGC104324, ZMYND1, ZNFN3A1, bA74P14.1 |
| G05 | Hs.522639 | NM_003173 | SUV39H1 | Suppressor of variegation 3-9 homolog 1 (Drosophila) | KMT1A, MG44, SUV39H |
| G06 | Hs.632120 | NM_016028 | SUV420H1 | Suppressor of variegation 4-20 homolog 1 (Drosophila) | CGI85, KMT5B, MGC118906, MGC118909, MGC21161, MGC703 |
| G07 | Hs.379466 | NM_003336 | UBE2A | Ubiquitin-conjugating enzyme E2A | HHR6A, RAD6A, UBC2 |
| G08 | Hs.730071 | NM_003337 | UBE2B | Ubiquitin-conjugating enzyme E2B | E2-17kDa, HHR6B, HR6B, RAD6B, UBC2 |
| G09 | Hs.99819 | NM_006447 | USP16 | Ubiquitin specific peptidase 16 | UBP-M |
| G10 | Hs.8015 | NM_012475 | USP21 | Ubiquitin specific peptidase 21 | MGC3394, USP16, USP23 |
| G11 | Hs.462492 | NM_015276 | USP22 | Ubiquitin specific peptidase 22 | KIAA1063, USP3L |
| G12 | Hs.113876 | NM_007331 | WHSC1 | Wolf-Hirschhorn syndrome candidate 1 | FLJ23286, KIAA1090, MGC176638, MMSET, NSD2, REIIBP, TRX5, WHS |
| H01 | Hs.534255 | NM_004048 | B2M | Beta-2-microglobulin | - |
| H02 | Hs.412707 | NM_000194 | HPRT1 | Hypoxanthine phosphoribosyltransferase 1 | HGPRT, HPRT |
| H03 | Hs.728776 | NM_012423 | RPL13A | Ribosomal protein L13a | L13A, TSTA1 |
| H04 | Hs.592355 | NM_002046 | GAPDH | Glyceraldehyde-3-phosphate dehydrogenase | G3PD, GAPD, MGC88685 |
| H05 | Hs.520640 | NM_001101 | ACTB | Actin, beta | PS1TP5BP1 |
| H06 | N/A | SA_00105 | HGDC | Human Genomic DNA Contamination | HIGX1A |
| H07 | N/A | SA_00104 | RTC | Reverse Transcription Control | RTC |
| H08 | N/A | SA_00104 | RTC | Reverse Transcription Control | RTC |
| H09 | N/A | SA_00104 | RTC | Reverse Transcription Control | RTC |
| H10 | N/A | SA_00103 | PPC | Positive PCR Control | PPC |
| H11 | N/A | SA_00103 | PPC | Positive PCR Control | PPC |
| H12 | N/A | SA_00103 | PPC | Positive PCR Control | PPC |

**Table B** – List of modulated genes comparing infected cells versus non-infected cells (control group) at 6h time-point.

| **RefSeq** | **Gene** | **Description** | **Symbol** | **2^-ΔC_t_** | | **Fold Up- or Down-Regulation** |
| --- | --- | --- | --- | --- | --- | --- |
|  |  |  |  | **Infected** | **Non infected** | **Infected /Non infected** |
| NM_014648 | DZIP3 | DAZ interacting protein 3, zinc finger | FLJ13327, FLJ57977, FLJ58022, FLJ58223, KIAA0675, UURF2, hRUL138 | 2,1E-03 | 1,3E-02 | **-6,42** |
| NM_019854 | PRMT8 | Protein arginine methyltransferase 8 | HRMT1L3, HRMT1L4 | 1,1E-05 | 5,2E-05 | **-4,84** |

**Table C** – List of modulated genes comparing infected cells versus non-infected cells (control group) at 12h time-point.

| **RefSeq** | **Gene** | **Description** | **Symbol** | **2^-ΔC_t_** | | **Fold Up- or Down-Regulation** |
| --- | --- | --- | --- | --- | --- | --- |
|  |  |  |  | **Infected** | **Non infected** | **Infected /Non infected** |
| NM_003160 | AURKC | Aurora kinase C | AIE2, AIK3, ARK3, AurC, STK13, aurora-C | 9,0E-04 | 2,5E-04 | **3,61** |
| NM_004824 | CDYL | Chromodomain protein, Y-like | CDYL1, DKFZp586C1622, MGC131936 | 3,1E-02 | 2,5E-03 | **12,43** |
| NM_001379 | DNMT1 | DNA (cytosine-5-)-methyltransferase 1 | AIM, CXXC9, DNMT, FLJ16293, MCMT, MGC104992 | 3,0E-01 | 8,8E-02 | **3,43** |
| NM_005933 | MLL | Myeloid/lymphoid or mixed-lineage leukemia (trithorax homolog, Drosophila) | ALL-1, CXXC7, FLJ11783, HRX, HTRX1, KMT2A, MLL, GAS7, MLL1A, TET1-MLL, TRX1 | 1,2E-01 | 1,8E-02 | **6,45** |

**Table D** – List of modulated genes comparing infected cells versus non-infected cells (control group) at 24h Activated time-point.

| **RefSeq** | **Gene** | **Description** | **Symbol** | **2^-ΔC_t_** | | **Fold Up- or Down-Regulation** |
| --- | --- | --- | --- | --- | --- | --- |
|  |  |  |  | **Infected** | **Non infected** | **Infected /Non infected** |
| NM_001379 | DNMT1 | DNA (cytosine-5-)-methyltransferase 1 | AIM, CXXC9, DNMT, FLJ16293, MCMT, MGC104992 | 9,1E-01 | 2,1E-01 | **4,27** |
| NM_003642 | HAT1 | Histone acetyltransferase 1 | KAT1 | 3,8E-03 | 3,6E-02 | **-9,40** |
| NM_024827 | HDAC11 | Histone deacetylase 11 | FLJ22237, HD11 | 4,0E-03 | 1,3E-03 | **3,08** |
| NM_012330 | KAT6B | K(lysine) acetyltransferase 6B | DKFZp313G1618, FLJ90335, KIAA0383, MORF, MOZ2, MYST4, qkf, querkopf | 3,1E-05 | 2,1E-04 | **-6,76** |
| NM_004586 | RPS6KA3 | Ribosomal protein S6 kinase, 90kDa, polypeptide 3 | CLS, HU-3, ISPK-1, MAPKAPK1B, MRX19, RSK, RSK2, S6K-alpha3, p90-RSK2, pp90RSK2 | 9,2E+02 | 5,0E-02 | **18253,39** |
| NM_031915 | SETDB2 | SET domain, bifurcated 2 | C13orf4, CLLD8, CLLL8, DKFZp586I0123, DKFZp761J1217, KMT1F | 1,4E+04 | 3,7E-02 | **384726,00** |

**Table E** – List of modulated genes comparing infected cells versus non-infected cells (control group) at 24h Non Activated time-point.

| **RefSeq** | **Gene** | **Description** | **Symbol** | **2^-ΔC_t_** | | **Fold Up- or Down-Regulation** |
| --- | --- | --- | --- | --- | --- | --- |
|  |  |  |  | **Infected** | **Non infected** | **Infected /Non infected** |
| NM_018489 | ASH1L | Ash1 (absent, small, or homeotic)-like (Drosophila) | ASH1, ASH1L1, FLJ10504, KIAA1420, KMT2H | 1,7E-02 | 9,7E-02 | **-5,74** |
| NM_001880 | ATF2 | Activating transcription factor 2 | CRE-BP1, CREB2, HB16, MGC111558, TREB7 | 2,9E-02 | 1,6E-01 | **-5,55** |
| NM_004217 | AURKB | Aurora kinase B | AIK2, AIM-1, AIM1, ARK2, AurB, IPL1, STK12, STK5, aurkb-sv1, aurkb-sv2 | 6,2E-02 | 3,5E-03 | **17,86** |
| NM_003160 | AURKC | Aurora kinase C | AIE2, AIK3, ARK3, AurC, STK13, aurora-C | 6,4E-04 | 2,6E-03 | **-4,03** |
| NM_004824 | CDYL | Chromodomain protein, Y-like | CDYL1, DKFZp586C1622, MGC131936 | 2,6E-02 | 1,1E-01 | **-4,24** |
| NM_000246 | CIITA | Class II, major histocompatibility complex, transactivator | C2TA, CIITAIV, MHC2TA, NLRA | 1,9E-02 | 1,1E-01 | **-5,92** |
| NM_020536 | CSRP2BP | CSRP2 binding protein | ATAC2, CRP2BP, KAT14, MGC15388, PRO1194, dJ717M23.1 | 1,0E-02 | 3,0E-02 | **-3,01** |
| NM_001379 | DNMT1 | DNA (cytosine-5-)-methyltransferase 1 | AIM, CXXC9, DNMT, FLJ16293, MCMT, MGC104992 | 9,1E-01 | 7,4E+00 | **-8,10** |
| NM_014648 | DZIP3 | DAZ interacting protein 3, zinc finger | FLJ13327, FLJ57977, FLJ58022, FLJ58223, KIAA0675, UURF2, hRUL138 | 3,9E-03 | 7,8E-02 | **-19,94** |
| NM_006709 | EHMT2 | Euchromatic histone-lysine N-methyltransferase 2 | BAT8, C6orf30, DKFZp686H08213, FLJ35547, G9A, KMT1C, NG36 | 1,7E-02 | 6,0E-02 | **-3,46** |
| NM_052911 | ESCO1 | Establishment of cohesion 1 homolog 1 (S. cerevisiae) | A930014I12Rik, CTF, ECO1, EFO1, ESO1, KIAA1911, MGC105022 | 9,4E-03 | 5,6E-02 | **-5,92** |
| NM_001017420 | ESCO2 | Establishment of cohesion 1 homolog 2 (S. cerevisiae) | 2410004I17Rik, EFO2, RBS | 1,1E-01 | 1,9E-03 | **61,50** |
| NM_032019 | HDAC10 | Histone deacetylase 10 | DKFZp761B039, HD10, MGC149722 | 2,3E-02 | 1,4E-01 | **-6,24** |
| NM_024827 | HDAC11 | Histone deacetylase 11 | FLJ22237, HD11 | 4,0E-03 | 4,6E-02 | **-11,40** |
| NM_001527 | HDAC2 | Histone deacetylase 2 | HD2, RPD3, YAF1 | 6,6E-04 | 5,6E-03 | **-8,48** |
| NM_006037 | HDAC4 | Histone deacetylase 4 | AHO3, BDMR, HA6116, HD4, HDAC-A, HDACA, KIAA0288 | 3,8E-02 | 2,4E-01 | **-6,32** |
| NM_005474 | HDAC5 | Histone deacetylase 5 | FLJ90614, HD5, NY-CO-9 | 6,5E-04 | 1,1E-02 | **-16,42** |
| NM_006044 | HDAC6 | Histone deacetylase 6 | FLJ16239, HD6 | 2,2E-02 | 8,8E-02 | **-3,90** |
| NM_001098416 | HDAC7 | Histone deacetylase 7 | DKFZp586J0917, FLJ99588, HD7A, HDAC7A | 8,6E-02 | 3,6E-01 | **-4,21** |
| NM_018486 | HDAC8 | Histone deacetylase 8 | HD8, HDACL1, RPD3 | 1,2E-02 | 4,1E-02 | **-3,46** |
| NM_178425 | HDAC9 | Histone deacetylase 9 | DKFZp779K1053, HD7, HD7b, HD9, HDAC, HDAC7, HDAC7B, HDAC9B, HDAC9FL, HDRP, KIAA0744, MITR | 2,9E-04 | 4,0E-03 | **-13,79** |
| NM_006618 | KDM5B | Lysine (K)-specific demethylase 5B | CT31, FLJ10538, FLJ12459, FLJ12491, FLJ16281, FLJ23670, JARID1B, PLU-1, PLU1, PUT1, RBBP2H1A | 8,2E-03 | 3,2E-02 | **-3,88** |
| NM_014663 | KDM4A | Lysine (K)-specific demethylase 4A | JHDM3A, JMJD2, JMJD2A, KIAA0677 | 3,2E-02 | 1,4E-01 | **-4,55** |
| NM_015061 | KDM4C | Lysine (K)-specific demethylase 4C | FLJ25949, GASC1, JHDM3C, JMJD2C, KIAA0780, bA146B14.1 | 1,5E-02 | 8,1E-02 | **-5,34** |
| NM_001080424 | KDM6B | Lysine (K)-specific demethylase 6B | JMJD3, KIAA0346 | 3,3E-02 | 2,2E-01 | **-6,77** |
| NM_021078 | KAT2A | K(lysine) acetyltransferase 2A | GCN5, GCN5L2, MGC102791, PCAF-b, hGCN5 | 4,8E-02 | 1,8E-01 | **-3,80** |
| NM_006388 | KAT5 | K(lysine) acetyltransferase 5 | ESA1, HTATIP, HTATIP1, PLIP, TIP, TIP60, cPLA2 | 1,8E-02 | 7,0E-02 | **-3,94** |
| NM_005933 | MLL | Myeloid/lymphoid or mixed-lineage leukemia (trithorax homolog, Drosophila) | ALL-1, CXXC7, FLJ11783, HRX, HTRX1, KMT2A, MLL, GAS7, MLL1A, TET1-MLL, TRX1 | 1,9E-02 | 9,0E-02 | **-4,79** |
| NM_170606 | MLL3 | Myeloid/lymphoid or mixed-lineage leukemia 3 | DKFZp686C08112, FLJ12625, FLJ38309, HALR, KIAA1506, KMT2C, MGC119851, MGC119852, MGC119853 | 9,3E-04 | 5,5E-03 | **-5,87** |
| NM_182931 | MLL5 | Myeloid/lymphoid or mixed-lineage leukemia 5 (trithorax homolog, Drosophila) | FLJ10078, FLJ14026, HDCMC04P, KMT2E, MGC70452 | 3,0E-03 | 1,6E-02 | **-5,42** |
| NM_001085487 | MYSM1 | Myb-like, SWIRM and MPN domains 1 | 2A-DUB, 2ADUB, DKFZp779J1554, DKFZp779J1721, KIAA1915, RP4-592A1.1 | 2,1E-02 | 1,4E-01 | **-6,64** |
| NM_032188 | KAT8 | K(lysine) acetyltransferase 8 | FLJ14040, MOF, MYST1, hMOF | 2,5E-02 | 1,1E-01 | **-4,44** |
| NM_007067 | KAT7 | K(lysine) acetyltransferase 7 | HBO1, HBOA, MYST2 | 3,6E-02 | 1,7E-01 | **-4,60** |
| NM_006766 | KAT6A | K(lysine) acetyltransferase 6A | MGC167033, MOZ, MYST3, RUNXBP2, ZNF220 | 8,3E-02 | 6,8E-01 | **-8,20** |
| NM_012330 | KAT6B | K(lysine) acetyltransferase 6B | DKFZp313G1618, FLJ90335, KIAA0383, MORF, MOZ2, MYST4, qkf, querkopf | 3,1E-05 | 1,8E-03 | **-57,88** |
| NM_003743 | NCOA1 | Nuclear receptor coactivator 1 | F-SRC-1, KAT13A, MGC129719, MGC129720, RIP160, SRC1, bHLHe42, bHLHe74 | 5,2E-04 | 5,7E-03 | **-10,99** |
| NM_014071 | NCOA6 | Nuclear receptor coactivator 6 | AIB3, ASC2, KIAA0181, NRC, PRIP, RAP250, TRBP | 9,9E-03 | 4,2E-02 | **-4,21** |
| NM_014397 | NEK6 | NIMA (never in mitosis gene a)-related kinase 6 | SID6-1512 | 9,4E-02 | 2,8E-01 | **-3,03** |
| NM_022455 | NSD1 | Nuclear receptor binding SET domain protein 1 | ARA267, DKFZp666C163, FLJ10684, FLJ22263, FLJ44628, KMT3B, SOTOS, STO | 4,2E-02 | 2,0E-01 | **-4,68** |
| NM_001535 | PRMT2 | Protein arginine methyltransferase 2 | HRMT1L1, MGC111373 | 1,7E-01 | 7,6E-01 | **-4,57** |
| NM_019854 | PRMT8 | Protein arginine methyltransferase 8 | HRMT1L3, HRMT1L4 | 2,8E-05 | 4,7E-04 | **-16,43** |
| NM_007212 | RNF2 | Ring finger protein 2 | BAP-1, BAP1, DING, HIPI3, RING1B, RING2 | 9,5E-03 | 3,1E-02 | **-3,28** |
| NM_019592 | RNF20 | Ring finger protein 20 | BRE1, BRE1A, FLJ11189, FLJ20382, KIAA2779, MGC129667, MGC129668, hBRE1 | 2,1E-03 | 8,4E-03 | **-4,04** |
| NM_004586 | RPS6KA3 | Ribosomal protein S6 kinase, 90kDa, polypeptide 3 | CLS, HU-3, ISPK-1, MAPKAPK1B, MRX19, RSK, RSK2, S6K-alpha3, p90-RSK2, pp90RSK2 | 9,2E+02 | 2,2E-01 | **4107,97** |
| NM_014712 | SETD1A | SET domain containing 1A | KIAA0339, KMT2F, Set1, Set1A | 4,5E-02 | 1,7E-01 | **-3,75** |
| NM_015048 | SETD1B | SET domain containing 1B | FLJ20803, KIAA1076, KMT2G, Set1B | 2,4E-02 | 1,4E-01 | **-5,70** |
| NM_014159 | SETD2 | SET domain containing 2 | FLJ16420, FLJ22472, FLJ23184, FLJ45883, FLJ46217, HBP231, HIF-1, HIP-1, HYPB, KIAA1732, KMT3A, SET2, p231HBP | 6,6E-02 | 3,9E-01 | **-5,91** |
| NM_017438 | SETD4 | SET domain containing 4 | C21orf18, C21orf27 | 7,7E-03 | 4,5E-02 | **-5,77** |
| NM_001080517 | SETD5 | SET domain containing 5 | DKFZp686J18276, FLJ10707, KIAA1757 | 3,4E-02 | 1,4E-01 | **-4,19** |
| NM_024860 | SETD6 | SET domain containing 6 | FLJ21148 | 7,2E-03 | 6,8E-02 | **-9,35** |
| NM_030648 | SETD7 | SET domain containing (lysine methyltransferase) 7 | FLJ21193, KIAA1717, KMT7, SET7, SET7, 9, SET9 | 9,5E-03 | 4,8E-02 | **-5,07** |
| NM_012432 | SETDB1 | SET domain, bifurcated 1 | ESET, H3-K9-HMTase4, KG1T, KIAA0067, KMT1E | 4,0E-02 | 2,2E-01 | **-5,41** |
| NM_031915 | SETDB2 | SET domain, bifurcated 2 | C13orf4, CLLD8, CLLL8, DKFZp586I0123, DKFZp761J1217, KMT1F | 1,4E+04 | 2,1E-01 | **68374,51** |
| NM_016028 | SUV420H1 | Suppressor of variegation 4-20 homolog 1 (Drosophila) | CGI85, KMT5B, MGC118906, MGC118909, MGC21161, MGC703 | 2,1E-02 | 1,8E-01 | **-8,61** |
| NM_003337 | UBE2B | Ubiquitin-conjugating enzyme E2B | E2-17kDa, HHR6B, HR6B, RAD6B, UBC2 | 8,8E-02 | 3,8E-01 | **-4,35** |
| NM_006447 | USP16 | Ubiquitin specific peptidase 16 | UBP-M | 9,3E-03 | 9,2E-02 | **-9,93** |
| NM_012475 | USP21 | Ubiquitin specific peptidase 21 | MGC3394, USP16, USP23 | 3,2E-02 | 1,3E-01 | **-4,10** |
| NM_012423 | RPL13A | Ribosomal protein L13a | L13A, TSTA1 | 1,0E+00 | 3,4E+00 | **-3,27** |

**Table F** – List of modulated genes comparing infected cells versus non-infected cells (control group) at 36h time-point.

| **RefSeq** | **Gene** | **Description** | **Symbol** | **2^-ΔC_t_** | | **Fold Up- or Down-Regulation** |
| --- | --- | --- | --- | --- | --- | --- |
|  |  |  |  | **Infected** | **Non infected** | **Infected /Non infected** |
| NM_005933 | MLL | Myeloid/lymphoid or mixed-lineage leukemia (trithorax homolog, Drosophila) | ALL-1, CXXC7, FLJ11783, HRX, HTRX1, KMT2A, MLL, GAS7, MLL1A, TET1-MLL, TRX1 | 8,4E-02 | 1,9E-02 | **4,30** |
| NM_024860 | SETD6 | SET domain containing 6 | FLJ21148 | 1,1E-03 | 3,0E-02 | **-27,33** |
