# Supplementary material for: Epigenetic Modulations in Activated Cells Early after HIV-1 Infection and Their Possible Functional Consequences
Source: PLoS One. 2015 Apr 13;10(4):e0119234. doi: 10.1371/journal.pone.0119234 (PMC4395311; doi:10.1371/journal.pone.0119234)
Supplement: S1 Methods — (DOC) [file pone.0119234.s004.doc]

**Supplemental Methods**

**Quantification of proviral loads *in vitro* infected samples**

HIV proviral loads were measured by qPCR using the HIV-1 integrase gene as target and the human CCR5 gene as normalizer. We extrapolated copy numbers from standard curves generated with plasmids harboring the CCR5 and the HIV integrase gene sequences, as described by Sharkey et al., 2000 and Komninakis et al., 2012. Briefly the reaction mixture consisted of , 20µL of Gene expression master mix (Life Technologies, Carlsbad, California, USA Applied Biosystems, Coutabouef, France) 2x, 0,75 μM of each oligonucleotide; 0,3μM of each probe, 5 μl of sample and DNAse RNAse –free water q.s.p 20µL. Sequences of primers and probes and cycling conditions are available at the above mentioned citations.

**Supplemental References**

Sharkey ME, Teo I, Greenough T, et al.: Persistence of episomal HIV-1 infection intermediates in patients on highly active anti-retroviral therapy. **Nat Med.** 6:76-81, 2000.

Komninakis SV, Santos DE, Santos C, Oliveros MP, Sanabani S,Diaz RS: HIV-1 proviral DNA loads (as determined by quantitative PCR) in patients subjected to structured treatment interruption after antiretroviral therapy failure. **J Clin Microbiol.** 50:2132-2133, 2012.
